# *AKR1B1*-dependent fructose metabolism enhances malignancy of cancer cells

**DOI:** 10.1038/s41418-024-01393-4

**Published:** 2024-10-15

**Authors:** Qing Zhao, Bing Han, Lu Wang, Jia Wu, Siliang Wang, Zhenxing Ren, Shouli Wang, Haining Yang, Michele Carbone, Changsheng Dong, Gerry Melino, Wen-Lian Chen, Wei Jia

**Affiliations:** 1 Center for Translational Medicine and Shanghai Key Laboratory of Diabetes Mellitus, Shanghai Sixth People’s Hospital Affiliated to Shanghai Jiao Tong University School of Medicine, Shanghai, 200233 China; 2 Mayo Clinic, 4500 San Pablo Rd S, Jacksonville, FL 32224 USA; 3 Department of Pharmacology and Pharmacy, University of Hong Kong, Hong Kong, China; 4 Cancer Institute, Longhua Hospital, Shanghai University of Traditional Chinese Medicine, Shanghai, 200032 China; 5 Cancer Biology Program, University of Hawaii Cancer Center, Honolulu, HI 96813 USA; 6 Department of Experimental Medicine, University of Rome “Tor Vergata”, 00133 Rome, Italy

**Keywords:** Cancer metabolism, Lung cancer

## Abstract

Fructose metabolism has emerged as a significant contributor to cancer cell proliferation, yet the underlying mechanisms and sources of fructose for cancer cells remain incompletely understood. In this study, we demonstrate that cancer cells can convert glucose into fructose through a process called the *AKR1B1*-mediated polyol pathway. Inhibiting the endogenous production of fructose through *AKR1B1* deletion dramatically suppressed glycolysis, resulting in reduced cancer cell migration, inhibited growth, and the induction of apoptosis and cell cycle arrest. Conversely, the acceleration of endogenous fructose through *AKR1B1* overexpression has been shown to significantly enhance cancer cell proliferation and migration with increased S cell cycle progression. Our findings highlight the crucial role of endogenous fructose in cancer cell malignancy and support the need for further investigation into *AKR1B1* as a potential cancer therapeutic target.

## Introduction

Cancer cells have a high demand for glucose to support their uncontrolled proliferation [1]. Our previous research has shown that cancer cells, such as acute myeloid leukemia (AML) cells, have enhanced fructose utilization under low glucose conditions and that blocking fructose utilization can inhibit AML cell proliferation and enhance the effectiveness of Ara-C treatment [2]. Multiple studies, including our own [1–4], have found that fructose is a preferred energy source for many types of cancer cells. These cells often express high levels of the fructose transporter SLC2A5/GLUT5 [5], allowing them to take up more fructose from the surrounding environment and promoting cancer growth. However, most dietary fructose is converted to glucose, glycogen, and organic acids in the intestine and liver [6]. As a result, fasting blood fructose levels are about 1000 times lower (~0.005 mM) than glucose levels (~5.5 mM) under normal physiological conditions [7]. Cancer cells alter their metabolism to support their malignant properties [8]. We thus hypothesized that this unique metabolic feature in cancer cells must arise from additional fructose sources and that increased fructose utilization conferred advantages over the use of glucose.

Recent studies have found that dietary fructose has been implicated in tumor promotion [9]. However, the mechanism that drives these pathologies by the endogenous fructose remains unclear. The polyol pathway, which is found in most somatic cells, converts about 3% of intracellular glucose to fructose under normal physiological conditions through a two-step process [10]. In the first step glucose is reduced to sorbitol using NADPH and the enzyme aldose reductase (AKR1B1). The second step involves the conversion of sorbitol to fructose through the action of sorbitol dehydrogenase (SORD) and the conversion of NAD^+^ to NADH.

The Warburg effect, extensively studied in cancer cells, is pivotal in fueling cancer’s uncontrolled growth and invasion. It has also been observed in non-cancerous cells that undergo rapid proliferation, such as T lymphocytes [11]. Although certain glycolytic intermediates can negatively regulate key metabolic enzymes, potentially leading to a reduction of the Warburg effect in cancer cells, it is probable that alternative pathways exist to sustain high levels of this metabolic phenotype. These alternative pathways are likely crucial for meeting the increased energy and building block demands of cancer cells. Thus, it is important to determine whether the polyol pathway is one of these alternative pathways.

Cell migration is critical for a variety of pathophysiological processes, such as development, tissue homeostasis, immune monitoring, and cancer metastasis [12]. Cancer cell migration can occur through two distinct modes: single-cell migration and collective migration [13]. Single-cell migration can be further classified into two subtypes, including ameboid migration and mesenchymal migration. Ameboid migration is characterized by low adhesion forces and occurs through either a high-contractility myosin II-dependent mode driven by membrane blebbing or a protrusion-based mode under low cell contractility [13]. On the other hand, mesenchymal migration involves epithelial-to-mesenchymal transition (EMT), a dedifferentiation process that enables cancer cells to acquire an invasive, chemoresistant phenotype, as well as cancer stem cell properties [14]. Cancer cells have an inherent plasticity that allows them to adjust their migration mode in response to their microenvironment [15]. To develop effective anti-metastasis therapy, it is important to target factors that are essential for all forms of cancer cell motility. These may include metabolic homeostasis, cytoskeletal dynamics, and cell contractility [13]. By comprehensively unveiling these critical factors, it is possible to disrupt cancer cell migration and potentially inhibit tumor malignancy.

This study aims at investigating the molecular mechanism by which cancer cells produce endogenous fructose and the role of this type of fructose in metabolic homeostasis and tumor malignancy.

## Results

### *AKR1B1* is expressed in various types of cancers and facilitates the intracellular supply of fructose for cancer cell metabolism

**Fig. 1:**
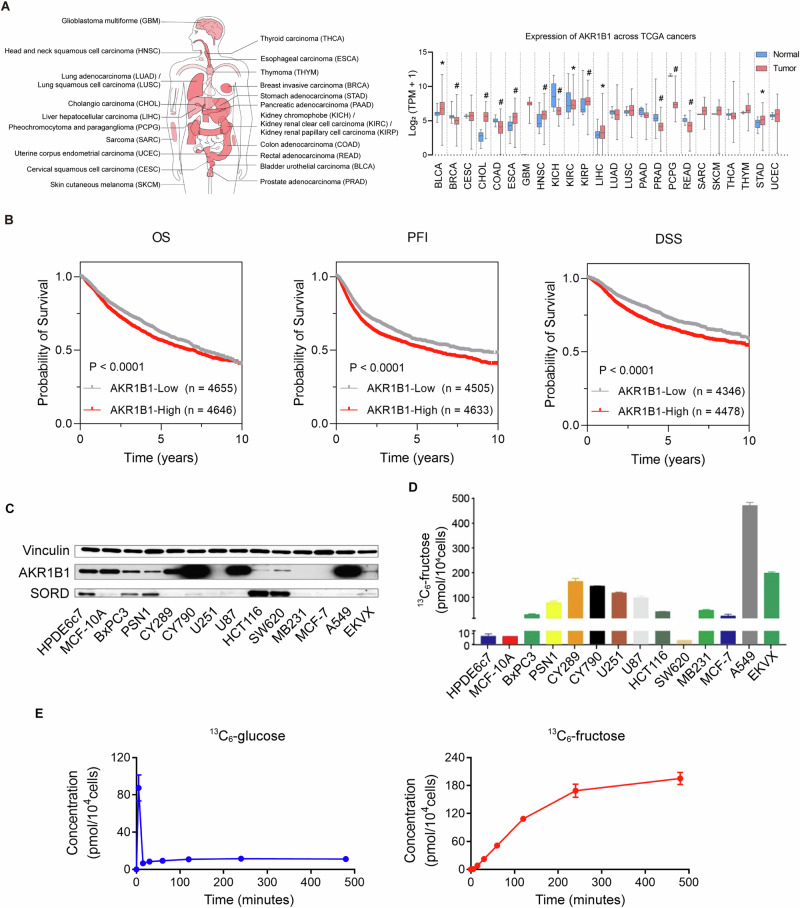
*AKR1B1* was widely expressed in multiple types of cancers and linked to active endogenous fructose biosynthesis in cancer cell lines. **A** (Left panel) Cancer types involved in the pan-cancer *AKR1B1* expression analysis of the right panel. (Right panel) *AKR1B1* expression in multiple cancer tissues and matched normal tissues of cancer patients from TCGA database. **B** Kaplan-Meier curves depicting OS, PFI and DSS of pan-cancer patients from TCGA database classified by low and high *AKR1B1* expression (RNA-seq). OS overall survival, PFI progression-free interval, DSS disease specific survival. **C** AKR1B1 and SORD were widely expressed in multiple cancer cell lines at protein level. **D** ^13^C- fructose production in different tumor cells. The cells were cultured in glucose-free DMEM medium containing 10% dialyzed FBS supplemented with 25 mM ^13^C-glucose for 24 h, harvested with cell scraping and followed by ^13^C-fructose quantification. **E** (Left panel) The ^13^C-glucose levels in A549 cells cultured in the same medium as (**D**) at distinct time points. (Right panel) The ^13^C-fructose production in A549 cells cultured in the same medium as (**D**) at distinct time points (n = 3). The error bars represent mean ± standard error of the mean (SEM). *: *t* test P < 0.05; #: *t* test P < 0.01.

The polyol pathway is the only known physiological pathway for converting glucose to fructose intracellularly. As the first step enzyme, we therefore investigated the clinical significance of *AKR1B1* expression in pan-cancer from human TCGA database. *AKR1B1* was widely expressed in multiple kinds of cancers (Fig. 1A), suggesting that the polyol pathway is an active and essential feature of cancer metabolism. What’s more, overall survival (OS), progression-free interval (PFI) and disease specific survival (DSS) curves of *AKR1B1* in pan-cancer from TCGA database showed that tumor patients with high expression of *AKR1B1* had worse prognosis (Fig. 1B). In addition, we conducted a bioinformatics analysis of other enzymes involved in the polyol pathway, such as ketohexokinase (KHK) and fructose bisphosphate aldolase (ALDOB). We found that these enzymes were also widely expressed in multiple types of cancers, as shown in Extended Data Fig. 1A. Consistent with our findings for *AKR1B1*, high expression of *KHK* was associated with poor progression-free interval (PFI) and disease-specific survival (DSS), while increased expression of *ALDOB* was linked to unfavorable overall survival (OS) and PFI, as demonstrated in Extended Data Fig. 1B, C. Then, we analyzed the expression of key enzymes involved in polyol pathway both at the gene and protein levels (Fig. 1C and Extended Data Fig. 2A, B) in cancer and control cell lines. We found that all cancer and control cell lines expressed varying levels of AKR1B1. As shown in Fig. 1D, incubation of 11 out of 12 cancer cell lines with fully labeled ^13^C-glucose resulted in the production of significant levels of ^13^C-fructose, indicating active endogenous fructose production from glucose in cancer cells. This was contrast to the low levels of ^13^C-fructose produced from ^13^C-glucose in control cell lines HPDE6c7 and MCF-10A (Fig. 1D). These results revealed that AKR1B1 was widely expressed in all these cell lines which was consistent with the observations in pan-cancer identified non-dietary sources of fructose produced by the polyol pathway as a result of cancer-mediated metabolic reprogramming. The highest endogenous fructose production was observed in the A549 cell line (Fig. 1D), a representative of human lung adenocarcinoma. Further analysis showed that A549 cells converted glucose to fructose rapidly, with intracellular ^13^C-fructose levels reaching a saturation point after 4 h of treatment with ^13^C-glucose (Fig. 1E). The high level of intracellular ^13^C-fructose suggested that the glucose taken up by A549 cells was largely converted to fructose for further metabolism. These findings demonstrated that cancer cells have a high demand for fructose and that the intracellular conversion of glucose to fructose is a new pathway to meet this demand.

To determine whether the glucose level affects the activation of the polyol pathway, we compared the expression of AKR1B1 in cancer cells of A549 and U87 cultured under different glucose levels. We found that glucose availability regulated the polyol pathway. Under high glucose (25 mM) concentrations, the cancer cells significantly increased their AKR1B1 expression levels compared to those grown in low glucose (2.5 mM) (Extended Data Fig. 2C).

**Fig. 2:**
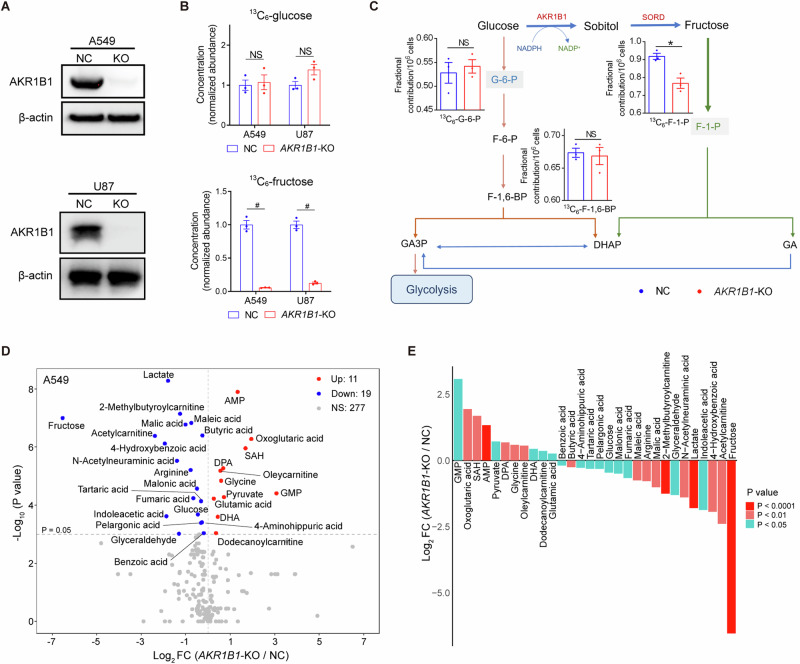
*AKR1B1*-mediated polyol pathway supplied endogenous fructose for cancer cell metabolism. **A** The comparison of AKR1B1 protein levels between control cancer cells and cancer cells with *AKR1B1*-KO. **B** (Upper panel) *AKR1B1*-KO had no significant influence on intracellular ^13^C-glucose content in tested cancer cells. (Lower panel) *AKR1B1*-KO blocked ^13^C-fructose production converted from exogenous ^13^C-glucose in tested cancer cells (n = 3). **C** A sketch illustrating the impact of *AKR1B1*-KO in A549 cells on glycolysis and polyol pathway. ^13^C-glucose was used as an isotope tracer (n = 3). **D** The volcano plot showing the perturbed metabolites by *AKR1B1*-KO in A549 cells (n = 3). **E** The bar graph displaying the differential metabolites triggered by *AKR1B1*-KO in A549 cells. These metabolites were ranked based on their fold change values and highlighted with different colors according to their P values. FC fold change. Data are represented as mean ± SEM. *: *t* test P < 0.05; #: *t* test P < 0.01; NS not significant.

To verify the role of the intracellular polyol pathway in glucose-fructose conversion, taking cancer incidence and malignancy into account, we blocked this pathway by deleting *AKR1B1* in A549 and U87 cells, which expressed high levels of *AKR1B1* (BxPC3 and HCT116 cells were used to overexpress *AKR1B1* subsequently for they expressed relatively low levels of this metabolic enzyme) (Extended Data Fig. 2D and Fig. 2A). By using ^13^C_6_-glucose as the carbon source in the culture media, we found that the deletion of *AKR1B1* did not significantly affect intracellular glucose levels, but effectively decreased the production of glucose-derived fructose (Fig. 2B). Notably, *AKR1B1* ablation did not disturb cell size of both A549 and U87 cells (Extended Data Fig. 2E). Additionally, *AKR1B1*-knockout (*AKR1B1-*KO) did not perturb the production of glucose-specific metabolites, glucose-6-phosphate (G-6-P) and fructose-1,6-bisphosphate (F-1,6-BP), but substantially downregulated the fructose-specific metabolite fructose-1-phosphate (F-1-P) in A549 cells (Fig. 2C). In U87 cells, both glucose- and fructose-specific metabolites were decreased upon *AKR1B1-*KO, with the fructose-specific metabolite F-1-P showing more significant fold change (Extended Data Fig. 2F). Collectively, these findings indicate that the *AKR1B1*-mediated polyol pathway significantly contributes to the intracellular production of fructose from glucose. It appears that the conversion from fructose to glucose is significant and that, once produced, the metabolism of fructose in cancer cells is independent of glucose metabolism. This process may provide materials more efficiently to meet the needs of rapidly proliferating tumor cells.

Targeted metabolomics profiling analysis showed that the intracellular content of fructose and lactate was decreased significantly by *AKR1B1*-KO in A549 (Fig. 2D, E) and U87 (Extended Data Fig. 2G) cells. Previous studies have shown that blocking fructose metabolism through *KHK* deletion can reduce ATP consumption and limit glycolytic activity, resulting in decreased lactate production [1]. However, in our study, we found that *AKR1B1* deletion did not affect intracellular ATP levels in either A549 or U87 cells, as shown in Extended Data Fig. 2H. Therefore, our results suggested that *AKR1B1* deletion may directly inhibit lactate synthesis. What’s more, the results of pathway enrichment analysis from differential metabolites implied that *AKR1B1*-KO significantly affected Warburg effect and urea cycle among the top 5 pathways both in A549 and U87 cells (Extended Data Fig. 2I-J). These results suggested that endogenous fructose, compared to exogenous fructose, may also play an extremely important role in tumor cells, and contributes to Warburg effect mainly through downstream metabolism by lactate production.

### *AKR1B1*-mediated fructose metabolism drives the excessive stimulation of glycolysis in cancer cells

**Fig. 3:**
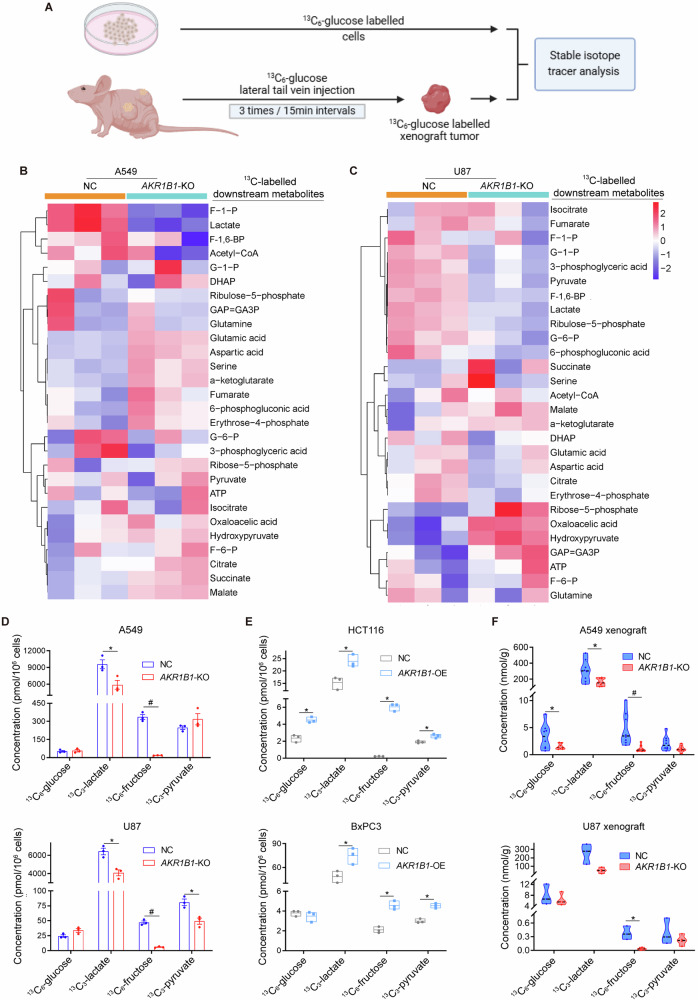
*AKR1B1*-mediated endogenous fructose metabolism contributed to glycolysis in cancer cells both in vitro and in vivo. **A** The strategy of establishment of in vitro and in vivo models and implementation of stable isotope tracer investigation in this study. Heatmaps showing the isotope tracing results by using^13^C-labeled glucose as a tracer in A549 (**B**) and U87 (**C**) cells with or without *AKR1B1*-KO. The cells were cultured in glucose-free DMEM medium containing 10% dialyzed FBS supplemented with 25 mM ^13^C-glucose for 24 h, harvested with trypsin and followed by metabolic flux analysis (n = 3). **D** Metabolite quantification for ^13^C-glucose, ^13^C-lactate, ^13^C-fructose, and ^13^C-pyruvate from A549 and U87 cells with or without *AKR1B1*-KO. The cell culture method is the same as described in (**B**) (n = 3). **E** Metabolite quantification for ^13^C-glucose, ^13^C-lactate, ^13^C-fructose, and ^13^C-pyruvate from HCT116 and BxPC3 cells with or without *AKR1B1* overexpression. The cell culture method is the same as described in (**B**) (n = 3). **F** Isotope tracing analysis by using ^13^C-labeled glucose as a tracer in A549 (n = 8) and U87 (n = 4) xenograft tumors with or without *AKR1B1*-KO. Data are represented as mean ± SEM. *: *t* test P < 0.05; #: *t* test P < 0.01.

The aforementioned results showed that blocking endogenous fructose metabolism resulted in a significant decrease in lactate production (Fig. 2D, E and Extended Data Fig. 2G). To further verify whether the reduced lactate was from glucose driven fructose metabolism both in vitro and in vivo, we performed stable isotope tracer investigation due to the diversity of lactate sources (Fig. 3A). First, in cancer cell line models in vitro, the heatmap of stable isotope tracer analysis showed changes in metabolites involved in glycolysis and mitochondrial respiration (Fig. 3B, C). We found fructose and glycolytic metabolites, such as lactate, were significantly decreased in *AKR1B1*-KO cells (Fig. 3D), while they were increased in *AKR1B1* overexpression cells (Fig. 3E and Extended Data Fig. 5A, B). These results suggested that blocking endogenous fructose production and metabolism by *AKR1B1*-KO dramatically inhibits glycolysis and the biosynthetic intermediates. Importantly, in vivo studies also confirmed that blocking glucose-derived fructose production and metabolism through *AKR1B1* deletion significantly decreases the levels of ^13^C-glucose-derived fructose and lactate in both A549 and U87 xenograft tumors (Fig. 3F). It was important to note that we identified all ^13^C-labeled downstream metabolites by analyzing the mass isotopologue distributions (MIDs) of each compound in both nontarget control (NC) and *AKR1B1*-KO cells from A549 (Extended Data Fig. 3) and U87 (Extended Data Fig. 4).

**Fig. 4:**
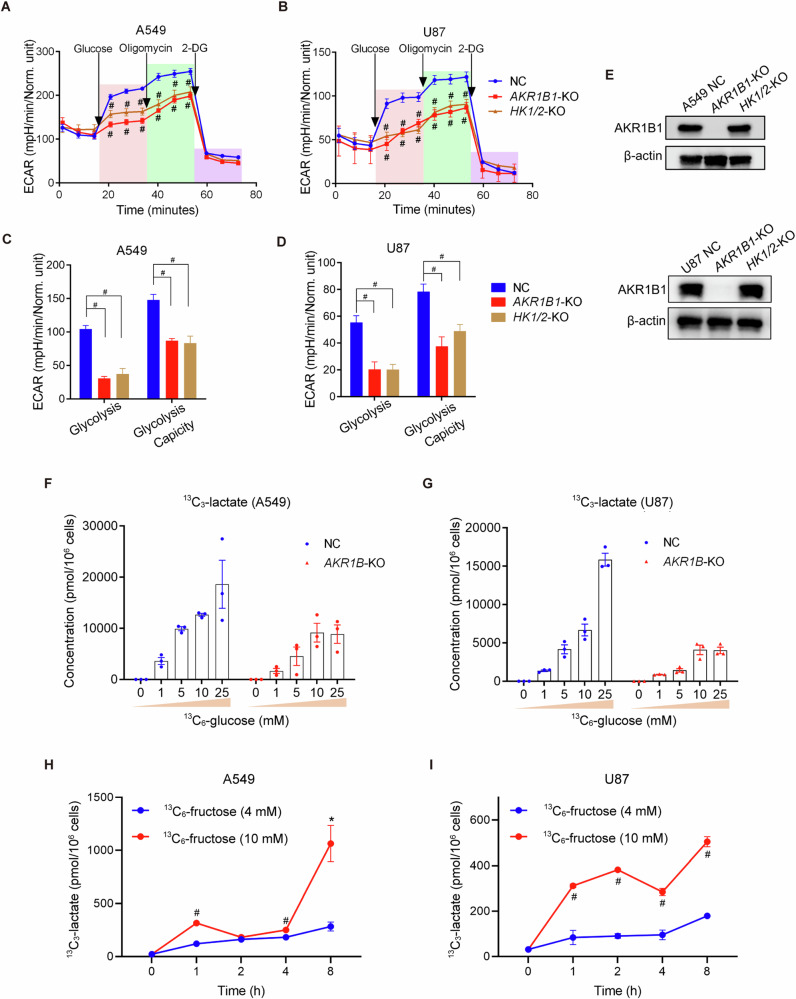
*AKR1B1*-mediated endogenous fructose metabolism fueled excessive stimulation of glycolysis. The Seahorse Cell Glycolysis Stress test showed the compromised glycolysis in *AKR1B1*-KO cells as well as *HK1/2*-KO cells of A549 (**A**) (n = 8) and U87 (**B**) (NC and *HK1/2*-KO group: n = 11, *AKR1B1*-KO: n = 7). Analysis of the glycolysis and glycolysis capacity in A549 (**C**) and U87 (**D**) cells by using the data in (**A**, **B**). **E** The comparison of AKR1B1 levels among control, *AKR1B1*-KO, and *HK1/2*-KO cells. Measurement of ^13^C-lactate production under distinct ^13^C-glucose concentrations in NC and *AKR1B1*-KO cells of A549 (**F**) and U87 (**G**) (n = 3). ^13^C-lactate production in A549 (**H**) and U87 (**I**) cells. The cells were cultured in glucose-free DMEM medium containing 10% dialyzed FBS supplemented with 4 mM or 10 mM ^13^C-fructose and harvested at different time points (0/1/2/4/8 h) for ^13^C- lactate quantification (n = 3). Data are represented as mean ± SEM. *: *t* test P < 0.05; #: *t* test P < 0.01.

We next investigated the role of intracellular fructose production in cancer cells. We hypothesized that the conversion of glucose to fructose could increase the diversity of intracellular carbon sources and enhance the glycolytic activity of cancer cells. To test this, we cultured cancer cells with impaired fructose production from glucose (*AKR1B1* deletion) in regular medium containing 25 mM glucose. We found that these cells had a drastically compromised glycolytic activity (Fig. 4A–D). In contrast, overexpression of *AKR1B1* in HCT116 and BxPC3 cells enhanced the glycolytic capacity (Extended Data Fig. 5C–F). Overall, these findings indicated that glucose-derived fructose production and metabolism is important to the glycolytic activity of cancer cells.

It is known that hexokinases, specifically hexokinase-1 (HK1) and hexokinase-2 (HK2), convert glucose to glucose-6-phosphate and initiate glycolysis (Fig. 2C). To further assess the role of intracellular fructose production in oncogenic glycolysis, we generated cancer cells with impaired glucose metabolism by deleting both *HK1* and *HK2* (Extended Data Fig. 5G). Firstly, we verified that *HK* knockout did not affect the expression of AKR1B1 (Fig. 4E). Then we compared the extracellular acidification rates (ECAR) by the Seahorse test in *AKR1B1-*KO or *HK1/2*-KO cells of A549 and U87 that were cultured in regular medium containing 25 mM glucose (Fig. 4A–D). The data showed that blocking either glucose or fructose metabolism resulted in a similar level of decrease in acidification in these cancer cells. Overall, these results suggested that the glycolytic activity of cancer cells is driven by the metabolism of both glucose and glucose-derived fructose.

Glucose metabolism is regulated by negative feedback mechanisms, such as the inhibition of phosphofructokinase (PFK) and hexokinase (HK) by glycolytic products like ATP and G-6-P [16]. In contrast, fructose can bypass these regulatory elements of classic glycolysis and continue to provide glyceraldehyde-3-phosphate (GA3P) for the later stages of glycolysis (Fig. 2C). Therefore, we hypothesized that cancer cells generate fructose from glucose to stimulate the Warburg effect constantly. To test this, we conducted the following experiments. Using media with different levels of glucose, we compared the output of lactate, a product of glycolysis, between cancer cells with *AKR1B1* deletion and control cells. The *AKR1B1-*KO cells were only able to metabolize glucose, while the control cells could metabolize both glucose and glucose-derived fructose. We found that lactate production reached saturation at low glucose concentrations in *AKR1B1*-KO cells (10 mM for A549 and U87 cells with *AKR1B1*-KO) (Fig. 4F, G). On the other hand, lactate production gradually increased in control cells as the glucose levels in the culture media were elevated and did not reach saturation even when the glucose concentration was as high as 25 mM (Fig. 4F, G). These results provided strong evidence that glucose-derived fructose production and metabolism is crucial for the stimulation of the Warburg effect in cancer cells.

To further investigate the metabolic fate of fructose and confirm its contribution to downstream glycolytic metabolites, we conducted an isotope tracing assay using ^13^C-fructose (labeled at all six carbons). Both A549 and U87 cells were cultured in glucose-free DMEM supplemented with 10% dialyzed FBS and varying concentrations of ^13^C-fructose. At different time points, we collected cells to measure downstream metabolites. We found that the production of ^13^C-lactate, a downstream metabolite derived from isotope-labeled fructose, was detectable in both A549 and U87 cells in a concentration- and time-dependent manner. The results demonstrated that tumor cells can absorb exogenous fructose and catabolize it to directly generate downstream metabolites, such as lactate, thereby contributing to increased glycolysis (Fig. 4H, I).

### *AKR1B1*-mediated fructose metabolism enhances cancer cell proliferation both in vitro and in vivo

**Fig. 5:**
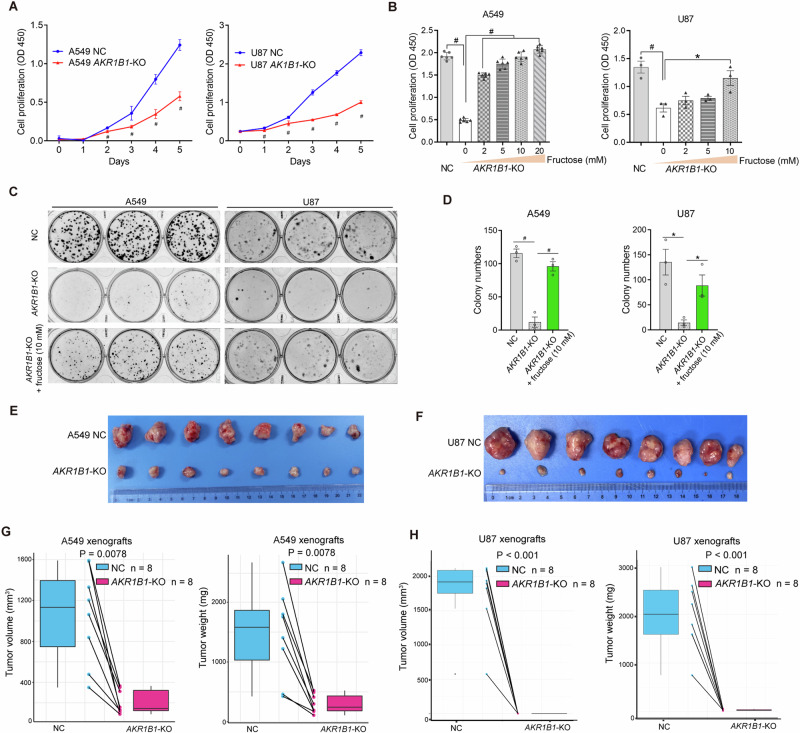
*AKR1B1*-mediated fructose production from glucose played an extremely important role in the proliferation of cancer cells both in vitro and in vivo*.* **A** Impaired proliferation for *AKR1B1*-KO cells as relative to NC cells. Cells were cultured in high glucose DMEM medium containing 10% FBS (n = 3). **B** Fructose supplementation recovered the proliferation of *AKR1B1*-KO cells. Cells were cultured in glucose-free DMEM medium containing 10% dialyzed FBS and 5 mM of glucose, supplemented with the indicated concentration of fructose for 4 days (A549 group: n = 6, U87 group: n = 3). **C**, **D** The images showing compromised colony formation in *AKR1B1*-KO cells, which was rescued by exogenous provision of fructose at 10 mM (**C**). Bar plots on the right side revealing the quantitative and statistical results (**D**). Cells were cultured in glucose-free DMEM medium containing 20% dialyzed FBS and 5 mM of glucose. Every 48 h, as indicated, the spent medium was exchanged with fresh media. After 14 days, the cells were fixed by 4% paraformaldehyde and stained with crystal violet for colony formation analysis (n = 3). *AKR1B1*-KO retarding the expansion of A549 (**E**) and U87 (**F**) xenograft tumors (n = 8). The negative impact of *AKR1B1*-KO on tumor volume and tumor weight of A549 (**G**) and U87 (**H**). Data are represented as mean ± SEM. *: *t* test P < 0.05; #: *t* test P < 0.01.

We then evaluated the impact of *AKR1B1*-mediated glucose-derived fructose production on the proliferation of cancer cells. In A549 and U87 cells with high baseline expression of *AKR1B1*, deleting this gene significantly inhibited cell proliferation (Fig. 5A) and colony formation (Fig. 5C–D). To confirm our findings, we used an *AKR1B1* inhibitor, epalrestat, to evaluate its effect on cell proliferation and colony formation. Our results showed that both A549 and U87 cells exhibited a significant decrease in proliferation when treated with 150 μM epalrestat, as demonstrated in Extended Data Fig. 6A, B. Furthermore, this inhibitor treatment dose-dependently inhibited colony formation of A549 and U87 cells, as shown in Extended Data Fig. 6C, D. These results were consistent with the findings of *AKR1B1*-KO. In addition, supplementing the medium with exogenous fructose was able to restore the impaired growth of cancer cells caused by *AKR1B1* deletion, as shown in Fig. 5B–D. In contrast, in HCT116 and BxPC3 cells with low baseline expression of *AKR1B1*, enforcing expression of this gene significantly increased cell growth and colony formation in regular medium (Extended Data Fig. 6E–H). These in vitro results demonstrated that glucose-derived intracellular fructose production and metabolism through the *AKR1B1*-mediated polyol pathway was critical for the neoplastic phenotypes of cancer cells.

We conducted in vivo studies to confirm the tumor-promoting role of endogenous fructose. Our results showed that blocking glucose-derived fructose production and metabolism through *AKR1B1*-KO significantly slowed xenograft tumor growth and reduced tumor weights in A549 and U87 cells (Fig. 5E–H). Overall, these data demonstrated that endogenous fructose production and metabolism from glucose through the *AKR1B1*-mediated polyol pathway was necessary for cancer cell growth in vivo.

### *AKR1B1*-mediated fructose metabolism augments cancer cell proliferation by inhibiting cell apoptosis and promoting S cell cycle progression

**Fig. 6:**
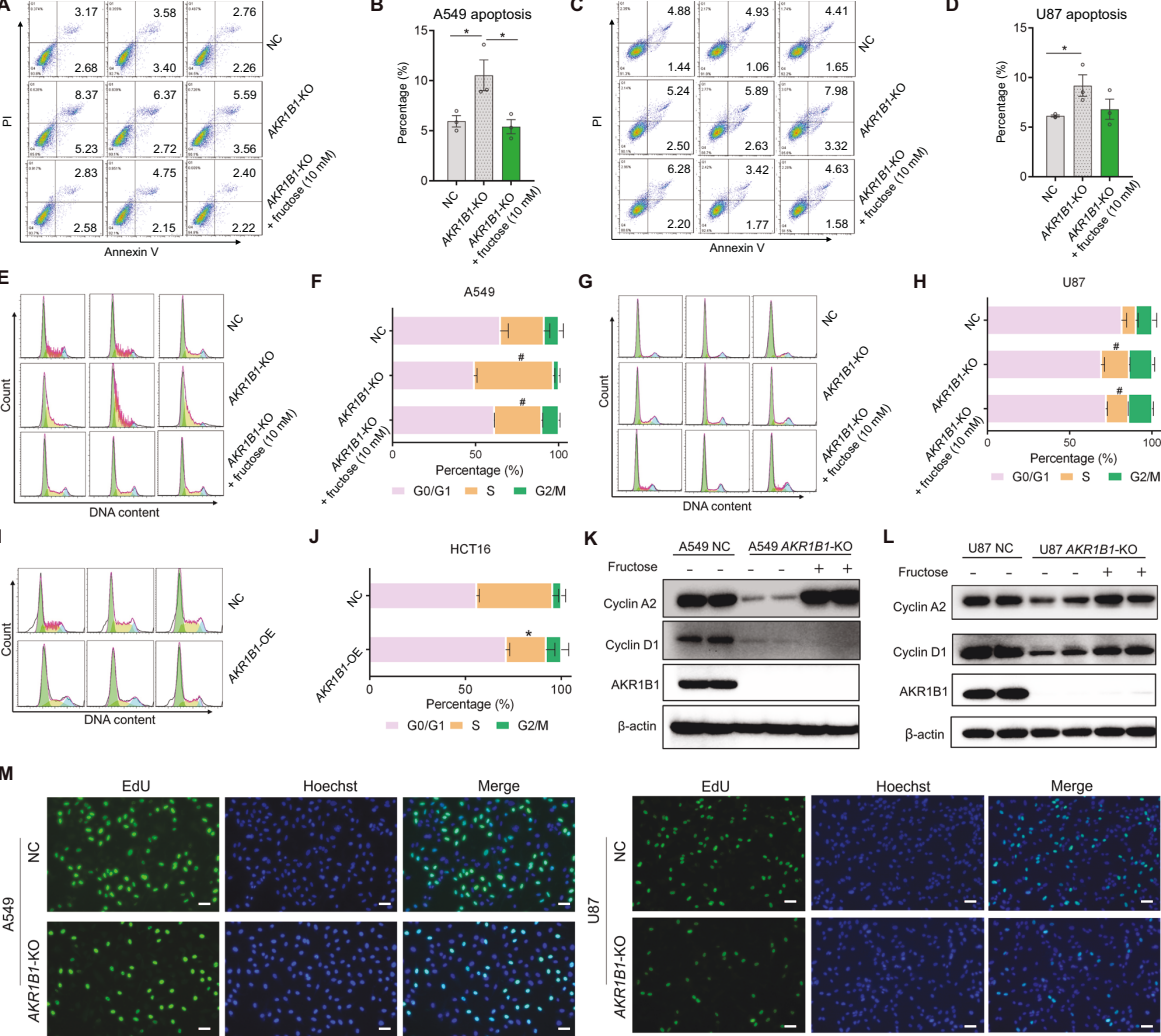
Glucose-derived fructose mediated by *AKR1B1* augmented cancer cell proliferation by hampering cell apoptosis and promoting S cell cycle progression. **A**–**D** *AKR1B1*-KO in A549 and U87 cells inducing cell apoptosis, while exogenous supplement of fructose reversing this phenotype. Quantitative and statistical analysis was exhibited by bar plots (**B**, **D**). Cells were cultured in glucose-free DMEM medium containing 10% dialyzed FBS and 5 mM of glucose for 48 h. Cells were then harvested for flow cytometry analysis (n = 3). **E**–**H** *AKR1B1*-KO in A549 and U87 cells eliciting S phase arrest, while exogenous supplement of fructose restoring S phase progression. Quantitative and statistical analysis was displayed by stacked bar plots (**F**, **H**). A549 and U87 cells were treated as mentioned in (**A**–**D**) and followed by flow cytometry for cell cycle analysis (n = 3). **I**–**J** *AKR1B1* overexpression in HCT116 cells expediting S phase progression. Cells cultured in high glucose DMEM medium containing 10% FBS were harvested for cell cycle analysis by flow cytometry (n = 3). **K**–**L** *AKR1B1*-KO in A549 and U87 cells downregulating the expression of cyclin A2 and cyclin D1, while exogenous supplement of fructose recovering the expression of these S phase marker proteins. A549 and U87 cells treated as described in (**A**–**D**) were analyzed by immunoblotting with indicated antibodies. **M** The EdU incorporation assay showed impaired DNA replication in *AKR1B1*-KO A549 and U87 cells. Scale bar is 100 μm. Data are represented as mean ± SEM. *: *t* test P < 0.05; #: *t* test P < 0.01.

To better understand the mechanism by which compromised fructose metabolism reduced viable cell proliferation of cancer cells, we examined the apoptosis and cell cycle profiles of A549 and U87 following treatment with or without fructose for 48 h. Flow cytometric analysis showed that blocking the conversion of glucose to fructose in cancer cells by knocking out the gene *AKR1B1* significantly increased cell apoptosis (Fig. 6A–D) and caused cell cycle arrest at the S phase (Fig. 6E–H). Similarly, epalrestat treatment also substantially elevated the apoptosis of A549 and U87 cells in a dose-dependent manner (Extended Data Fig. 7A, B). Moreover, epalrestat administration triggered overt S phase arrest of cancer cells (Extended Data Fig. 7C-D). Overexpression of *AKR1B1*, on the other hand, significantly fostered S phase progression (Fig. 6I, J) in regular culture medium containing 25 mM glucose. Supplementing the culture medium with exogenous fructose reversed these effects caused by *AKR1B1*-KO (Fig. 6A–H). Furthermore, blocking the generation of glucose-derived fructose through *AKR1B1*-KO significantly decreased the expression of cell cycle progression markers, such as cyclin A2 and cyclin D1, in A549 (Fig. 6K) and U87 cells (Fig. 6L). By contrast, fructose supplementation predominantly restored the expression of cyclin A2, a S phase marker protein, both in A549 and U87 cells with *AKR1B1* deletion. Taken together, these results suggested that endogenous fructose produced from glucose promotes cancer cell growth by boosting cell survival and S phase cell cycle progression.

Next, we investigated the underlying mechanism of S phase arrest elicited by *AKR1B1-*KO. We observed that the loss of *AKR1B1* dramatically restrained the incorporation of 5-ethynyl-2’-deoxyur-idine (EdU) into the nucleus of the cancer cells with impaired endogenous fructose metabolism. This indicated that the deletion of *AKR1B1* induces S phase arrest, potentially by blocking DNA replication (Fig. 6M).

### *AKR1B1*-mediated fructose metabolism enhances cancer cell migration via RhoA-ROCK2 pathway

**Fig. 7:**
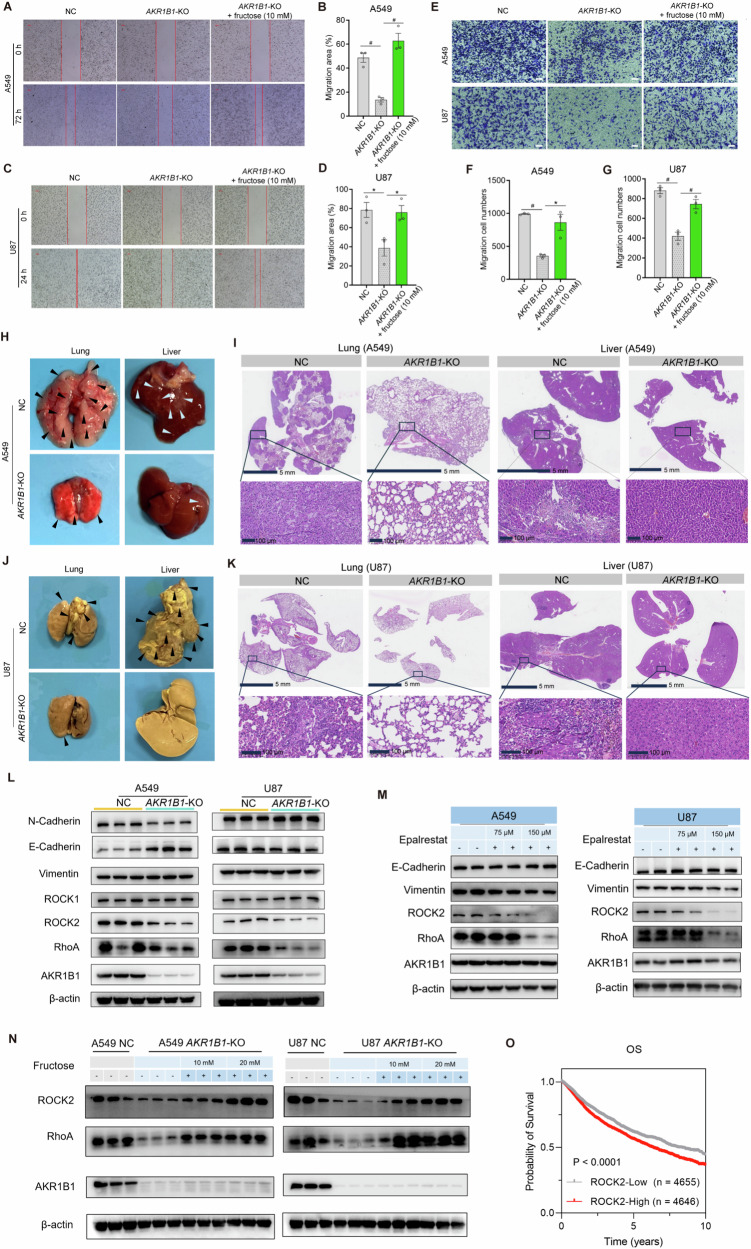
*AKR1B1*-mediated fructose production from glucose enhanced cancer cell migration via RhoA-ROCK2 pathway. **A**–**D** *AKR1B1*-KO in A549 and U87 cells slowing cell migration in the wound healing assay, while exogenous fructose restoring this behavior (n = 3). The wound healing assay was performed by using A549 and U87 cells cultured in glucose-free DMEM medium containing 2% dialyzed FBS and 5 mM of glucose. The spent medium was exchanged every day. **E**–**G** *AKR1B1*-KO in A549 and U87 cells impairing cell migratory capability in the transwell assay, while exogenous fructose restoring this capability (**E**). Quantitative and statistical analysis was manifested by bar plots for A549 (**F**) and U87 (**G**). In the transwell assay, cells were seeded in the upper chambers in glucose-free DMEM medium. The lower chamber was filled with glucose-free DMEM medium containing 10% dialyzed FBS and 5 mM of glucose (n = 3). Scale bar is 100 μm. **H**–**K** The negative influence of *AKR1B1-*KO on the formation of A549 (**H**–**I**) and U87 (**J**–**K**) metastatic nodules in the lungs and livers. The experimental metastasis mouse model was established through venous inoculation of corresponding cancer cells. The metastatic nodules of A549 (**I**) and U87 (**K**) cells were reflected by hematoxylin and eosin staining. **L** Comparative analysis of biomarker proteins involved in EMT and RhoA signal pathway between NC and *AKR1B1-*KO cells. **M** The impact of treatment of an AKR1B1 inhibitor, epalrestat, on the expression of biomarker proteins involved in EMT and RhoA signal pathway in A549 and U87 cells. **N** The rescue effect of exogenous fructose supplementation on the expression of biomarker proteins involved in EMT and RhoA signal pathway in *AKR1B1-*KO cells. **O** Kaplan-Meier curves of pan-cancer patients from TCGA database with low and high *ROCK2* mRNA expression. Data are represented as mean ± SEM. *: *t* test P < 0.05; #: *t* test P < 0.01.

We further investigated the impact of *AKR1B1*-mediated fructose production on the migration capacity of cancer cells. Deletion of *AKR1B1* significantly inhibited cancer cell migration, as demonstrated by both wound healing (Fig. 7A–D) and transwell assays (Fig. 7E–G). The impaired migration capacity caused by *AKR1B1*-KO was reversed by supplementation with fructose (Fig. 7A–G). Consistent results were observed in cells overexpressing *AKR1B1*, as enforced expression of this gene augmented the migration ability of tumor cells of HCT116 and BxPC3 (Extended Data Fig. 7E–H). Moreover, A549 and U87 cells with impaired endogenous fructose metabolism exhibited decreased metastasis to the lung or liver tissues (Fig. 7H–K). Notably, NC A549 cells exhibited a tendency to metastasize to the lung, while NC U87 cells showed a preference for liver metastasis.

Ameboid migration, a part of the epithelial-mesenchymal spectrum, is sustained by high levels of RHO-ROCK-driven Myosin II activity [17]. EMT is a dedifferentiation process that cancer cells undergo to acquire an invasive, chemoresistant phenotype, as well as properties of cancer stem cells [14]. We then conducted the following assays to ascertain the mechanism by which AKR1B1-mediated polyol pathway promoted cancer cell migration. We noticed that *AKR1B1* deletion remarkably downregulated the expression of RhoA and ROCK2 in both A549 and U87 cells (Fig. 7L). Of note, *AKR1B1* ablation gave rise to different influence on the expression of EMT markers, including E-cadherin and N-cadherin, between these two cell lines (Fig. 7L). Concordantly, treatment with an AKR1B1 inhibitor epalrestat also declined the expression of RhoA and ROCK2, but not E-cadherin and vimentin in these cell lines (Fig. 7M). These results indicated that *AKR1B1*-KO could suppress RhoA-ROCK2 pathway. By contrast, fructose addition strikingly recovered the expression of RhoA and ROCK2 in A549 and U87 cells with *AKR1B1*-KO in a dose-dependent manner (Fig. 7N). Intriguingly, high expression of ROCK2 predicted worse OS in pan-cancer patients from TCGA database (Fig. 7O).

In conclusion, these findings suggested that *AKR1B1* may play a crucial role in promoting tumor metastasis through activation of the RhoA-ROCK2 pathway. Previous study has reported that the RhoA-ROCK2 pathway predominantly mediates the single-cell metastasis mode in tumor cells [15]. Thereby, it is reasonable to postulate that *AKR1B1* may promote metastasis by facilitating the detachment of tumor cells.

## Discussion

Glucose has long been recognized as a primary metabolic fuel for cancer cell glycolysis [18, 19]. However, recent research, including ours, has shown that exogenous fructose can also serve as a carbon source for cancer cells. In this study, we discovered that the conversion of glucose to fructose is highly active in various cancer cells. This metabolic feature suggests that cancer cells have a strong reliance on fructose, while also providing cancer cells with diverse intracellular carbon sources. We identified that the *AKR1B1*-mediated polyol pathway, which is highly active in cancer cells, is a key mechanism for producing fructose from glucose. Through in vitro and in vivo isotope tracing assays, we demonstrated that the metabolism of glucose-derived intracellular fructose through the *AKR1B1*-mediated polyol pathway might be an alternative pathway of Warburg effect when glycolysis was regulated by negative feedback of products.

Cancer cells often overexpress glucose transporters such as SLC2A1/GLUT1 and SLC2A3/GLUT3 to take in large amounts of extracellular glucose to support their high rates of proliferation [20–22]. However, glucose metabolism is a tightly controlled process in which the activities of two rate-limiting enzymes, PFK and HK, can be negatively inhibited by glycolytic products such as ATP and glucose 6-phosphate, which limits glycolysis and results in a shortage of building blocks and energy for cancer cells [16, 23]. In this study, we found that cancer cells convert large amount of glucose to fructose. The metabolism of this endogenous fructose contributes to the Warburg effect in cancer cells, providing excess amounts of glycolytic metabolites for biomass and lactate production [24]. Blocking either glucose or fructose metabolism resulted in the decrease of glycolysis in these cancer cells. Thus, the aerobic glycolysis in cancer cells is likely fueled by a combination of glucose and glucose-derived fructose metabolism. Although *HK1* and *HK2* deletion did not indicate a complete blockade of glucose metabolism due to the limitation of technical conditions, it could still support our opinion. While the polyol pathway is not specific to cancer cells, we hypothesize that it has a significant impact on all rapidly growing cells, including activated immune cells, germinal cells, and stem cells. Overall, our findings implied that fructose metabolism might be essential to sustain high levels of the Warburg effect in cancer cells.

The polyol pathway is not crucial for energy metabolism in normal cells and inhibiting *AKR1B1* activity could be a viable and safe cancer treatment approach. This study found that high glucose levels enhance *AKR1B1* expression and may explain the elevated cancer risk in diabetic patients [25]. The polyol pathway is activated in diabetics to lower blood glucose levels and contributes to diabetic complications, including tissue damage and malignant cell transformation. The intermediate molecule in the polyol pathway, sorbitol, cannot cross cell membranes and its over accumulation causes microvascular damage to the retina, kidney, and nerves. There is evidence linking increased fructose levels to cancer malignancy, suggesting the polyol pathway’s activation may increase cancer risk in diabetics. Increased blood glucose activates the polyol pathway providing damaged cells with additional nutrient support to facilitate their malignant transformation. Clinical trials are testing AKR1B1 inhibitors for diabetic complications [26, 27] and these compounds may have potential as direct cancer treatments and preventive treatments for diabetic patients.

This study reveals a significant association between *AKR1B1*-mediated endogenous fructose metabolism, cell cycle progression, and cell migration. However, the underlying mechanisms are not yet fully understood. Previous studies have reported that changes in intracellular metabolism can directly affect the activity of cell cycle regulators and signaling pathways, thereby impacting cell cycle control [28]. For example, the TCA cycle is hyperactive in G1 phase, while glycolysis is aberrantly provoked in S phase in mammalian cells [29]. Our study highlights the importance of endogenous fructose, which is converted from glucose, on glycolytic activity. This suggests that endogenous fructose metabolism may promote S cell cycle progression by stimulating glycolysis. Additionally, our previous findings have shown that exogenous fructose metabolism can activate mTORC1 signaling, which promotes S cell cycle progression [3, 30]. Therefore, endogenous fructose metabolism may also have the potential to activate mTORC1 signaling, thus facilitating S phase progression. Moreover, mTORC1 can stimulate RhoA signaling in cancer cells, which can foster tumor metastasis [31]. Therefore, endogenous fructose metabolism may also promote tumor metastasis via the mTORC1-RhoA pathway. However, the exact mechanisms by which *AKR1B1*-mediated endogenous fructose metabolism governs cell cycle progression and cancer cell migration require further investigation.

Metastasis remains the leading cause of cancer-related mortality, despite advancements in diagnostic processes and cytotoxic therapies that have improved overall cancer survival rates [13]. Therefore, there is a continued need for novel approaches that specifically target metastasis. When faced with metabolic challenges, invading tumor cells can undergo bioenergetic adaptations to ensure cell survival and sustained migration. Hypoxia and nutrient deprivation can induce plasticity in invasion programs, leading to processes such as EMT, mesenchymal-to-ameboid transition, and collective-to-ameboid transition [32]. Understanding the intricate crosstalk between bioenergetic pathways and cell migration will aid in identifying potential intervention points to disrupt tumor cell dissemination and metastasis.

Metabolic reprogramming and invasion mechanisms are closely intertwined in cancer cells. These cells rely on energy metabolism programs to adapt to metabolic stress caused by toxic byproducts, enabling their survival and migration. The ability to rewire metabolism and utilize diverse energy sources is essential for sustaining migration. Moreover, cancer cells can dynamically adjust their migration mode, facilitating rapid invasion, making it a potential therapeutic target. Our findings confirm the significant role of *AKR1B1* in promoting invasion and metastasis by activating the RhoA-ROCK2 signaling pathway.

In summary, cancer cells utilize the polyol pathway to produce fructose and gain more energy and biomass than they would from an equivalent amount of glucose. Fructose metabolism in cancer cells leads to increased lactate and metabolic intermediates for biomass, contributing significantly to the Warburg effect. Additionally, *AKR1B1*-mediated endogenous fructose metabolism regulates proliferation and ameboid migration through the cyclin and RhoA-ROCK2 signaling pathways, ultimately increasing tumor malignancy. These findings suggest the potential for *AKR1B1* as a promising therapeutic target.

### Limitations of the study

Limitations of the study include the need for more extensive investigations into the role of *AKR1B1* in promoting cancer growth across various cancer types, beyond lung adenocarcinoma and glioblastoma, to determine its universality. Additionally, the study did not consider other potential functions of *AKR1B1* beyond fructose metabolism, which could impact the metabolic phenotype of tumor cells. Further clarification is needed to understand the reasons behind cancer cells’ induction of *AKR1B1*-mediated fructose synthesis from glucose as a substrate.

## Methods

### Cell culture

The A549, HCT116, U251, MCF7, and MDA-MB-231 cell lines were obtained from the National Cancer Institute and the University of Hawaii Cancer Center. The BxPC3, PSN1, and U87 cell lines were purchased from Sigma-Aldrich. The HPDE6c7 and MCF-10A cell lines were obtained from the American Type Culture Collection (ATCC). All the cell lines were cultured and maintained in Dulbecco’s modified Eagle’s medium (DMEM) with 10% fetal bovine serum (FBS) at 37°C and 5% CO_2_.

### Gene knock out by CRISPR/Cas9

We used Dharmacon Edit-R All-in-one Lentiviral and Edit-R CRISPRa Lentiviral systems to create gene knock out (KO) cell lines. The sgRNA sequences are as follows: *AKR1B1* forward, 5ʹ- CACCGTCAGGTCGCTGAGTGTCTTC-3ʹ and reverse, 5ʹ- AAACGAAGACACTCAGCGACCTGAC-3ʹ; *HK1* forward, 5ʹ- CACCGCAGAGCTTACCGATTCTCGC-3ʹ and reverse, 5ʹ- AAACGCGAGAATCGGTAAGCTCTGC-3ʹ; *HK2* forward, 5ʹ- CACCGTGACCACATTGCCGAATGCC-3ʹ and reverse, 5ʹ- AAACGGCATTCGGCAATGTGGTCAC-3ʹ. Retroviral particles were produced by co-transfection of 293 T cells with PMD_2_ and PsPAX_2_ packaging plasmids using polyethylenimine (PEI) as a transfection reagent (3:1 mass ratio of PEI:DNA). Stable cell lines were created by infecting the transfected cells according to the manufacturer’s instruction.

### Overexpression of *AKR1B1*

To overexpress *AKR1B1*, the *AKR1B1* open reading frame (ORF) was amplified from A549 cDNA and cloned into the retroviral vector Plvx-EF1α-AcGFP. Retroviral particles were produced by co-transfecting 293 T cells with TAT, PMD2, and PsPAX2 packaging plasmids using xfect Polymer as a transfection reagent in 6 mL Optim-EME (Gibco: 31985070). After 48 h, the virus-containing medium was collected, filtered through a 0.45 μm filter, aliquoted, and stored at –80 °C. To induce overexpression, the retrovirus-containing supernatant was added to HCT116 and BxPC3 cells grown in normal culture medium containing polybrene transfection reagent at a concentration of 10 μg/mL. The transduced cells were labeled with GFP and selected with puromycin at a concentration of 2 μg/mL. The level of overexpression was assessed by western blotting (WB).

### Western blotting

Cells for protein analysis were collected and total proteins were extracted by RIPA buffer with phosphatase and protease inhibitors. Protein content in lysates was determined by using the BCA Protein Assay Kit (Thermo Fisher Scientific). Protein samples were separated by 10% SDS-PAGE and transferred to PVDF membranes (Millipore, Bedford, MA, USA). The transfer-ready membrane was then blocked for one hour in TBS-T containing 5% nonfat milk at room temperature, followed by overnight incubation with primary antibody at 4°C. The anti-AKR1B1 antibody (1:1,000, Abcam, ab62795), anti-SORD antibody (1:1,000, Abcam, ab185705), anti-HK1 antibody (1:1,000, Abcam, ab150423), anti-HK2 antibody (1:1,000, Abcam, ab209847), anti-Cyclin A2 antibody (1:1,000, CST, 67955 T), anti-Cyclin D1 antibody (1:1,000, CST, 55506 T), anti-Vimentin antibody (1:1,000, CST, 5741 T), anti-N-Cadherin antibody (1:1,000, CST, 13116 T), anti-E-Cadherin antibody (1:1,000, CST, 3195 T), anti-RhoA antibody (1:1,000, CST, 22117 T), anti-ROCK1 antibody (1:1,000, CST, 4035 T), anti-ROCK2 antibody (1:1,000, Abcam, ab125025), anti-Vinculin antibody (1:1,000, Abcam, ab91459), and anti-beta actin antibody (1:1,000, Abcam, ab8227) respectively. A horseradish peroxidase-conjugated anti-rabbit secondary antibody was used at a 1:5,000 dilution. The bands were visualized using the enhanced chemiluminescence kit (Millipore) and protein bands were quantified with the Molecular Imager ChemiDoc XRS System (Bio-Rad Laboratories, Hercules, CA, USA).

### RT-PCR

RNA extraction and reverse transcription were performed by TRIzol (Thermo Fisher Scientific) and iScript™ cDNA Synthesis Kit (Bio-Rad Laboratories) according to the manufacturer’s instructions. PCR primer sequences were as follows. Primers for *AKR1B1* PCR: TACTCAGCTACAACAGGAACTG, AGGCAAGAAACACAG GTATAGG, probe: TTGTTGAGCTGTACCTCCCACAAGG; Primers for *SORD* PCR: ACTCCAGAGCCAAAAGAGC, CATCCTCAGCAAGACCTCAT, probe: AGGATAGTTCTCCAGGCGCAAGTC; AATTTCAC. We performed real-time PCR with the iTaq™ Universal Probes Supermix (Bio-Rad Laboratories) by Lightcycle 480 (Roche Molecular Systems).

### Seahorse extracellular flux assays

The experiments were performed on an Agilent Seahorse XF96 bioanalyzer following the manufacturer’s instructions. Gene-modified cancer cells were plated onto XF96 microplates (A549: 8000 cells/well, U87: 2 × 10^4^ cells/well). Before the assay, cells were maintained in serum-free HuMEC Ready Medium. The Seahorse XF Glycolysis Stress Test Kit was used to measure cell glycolysis, with three injections: (1) 10 mM glucose; (2) 1 μM oligomycin; and (3) 50 mM 2-DG.

### Cell proliferation assay

All gene-modified cancer cells were plated at 3000 cells/well in DMEM Medium with 10% FBS followed by the culture at 37 °C under 5% CO_2_. A cell proliferation assay was performed by using the Cell Counting Kit-8 (CCK-8) (Dojindo Molecular Technologies) after the indicated time point of culture. 10 μL of the CCK8 dye was added to each plate well and then incubated for one hour at 37 °C under 5% CO_2_. The optical density (OD) was then read at 450 nm directly using a microplate reader (MolecularDevices, SpectraMax i3x). For fructose supplementation experiments, glucose free DMEM and dialyzed FBS were used to exclude the influence of sugars in the culture medium.

### Colony formation assay

A549 (300 cells/well) or U87 (500 cells/well) cells were seeded into 6-well plates and cultured at 37 °C under 5% CO_2_ for the colony formation assay. Two weeks later, colonies were fixed with paraformaldehyde, followed by staining with crystal violet (0.5%, sigma). Finally, colonies were photographed with and counted using Image J.

### Wound healing and cell migration assays

For wound healing assay, U87 or A549 cells were seeded into a 6-well plate (5 × 10^5^ cells/well) one day in advance. Scratches were performed with a sterile 200 μL pipette tip held perpendicular to the plate bottom. After several PBS washes to remove floating cells, the cells were cultured in a DMEM medium with 1% FBS for 24 h. The wound areas were recorded at 0 and 24 h using a microscope (Nikon, Eclipse TS2R). The images of wound healing data were analyzed by ImageJ.

The transwell chambers (Costar, 3422) with 8 μm pores were utilized for cell migration assay. Briefly, cells (5 × 10^4^ cells per well) were seeded in the upper chambers with serum-free-DMEM medium, while the lower chamber was supplemented with 500 μl DMEM medium containing 10% FBS. Following incubation at 37 °C with 5% CO_2_ for 24 h, migration cells were fixed with 4% formaldehyde for 15 min and stained with 0.5% crystal violet for 10 min. Subsequently, the migrating cells were counted under the microscope in three random microscopic fields.

### Cell cycle and apoptosis assay

Cell apoptosis was examined by YF®488-Annexin V and PI Apoptosis Kit (US Everbright Inc, Y6002), and cell cycle was examined by Cell Cycle and Apoptosis Analysis Kit (US Everbright Inc, C6031) according to the manufacturer’s instructions. The samples were immediately analyzed by flow cytometry (Beckman), and the resulting flow cytometry data were processed using FlowJo software. Three technical replicates were performed.

### DNA replication of cells assay

A549 or U87 cells were seeded into a 12-well plate at a density of 1 × 10^5^ cells per well one day in advance. After incubating with 10 μM 5-ethynyl-2′-deoxyuridine (EdU) (Beyotime, C0071S) at 37 °C with 5% CO_2_ for one hour, the cells were fixed by 4% paraformaldehyde for 15 min and subsequently permeabilized with 0.03% Triton X-100 for 10 min at room temperature. Finally, a BeyoclickTM EdU Cell Proliferation Kit with Alexa Fluor 488 (Beyotime, C0071S) was employed for EdU incorporation assay, following the manufacturer’s instruction.

### ATP assay

The concentration of ATP in A549 and U87 cells was detected using the ATP Assay Kit (Beyotime, S0026), based on the manufacturer’s instructions. The analysis was performed immediately using a multimode microplate reader (MolecularDevices, SpectraMax i3x).

### Subcutaneous Xenograft tumor model

The mice used for animal experiments were thymic BALB/c nude mice (4–6 weeks old, female) from Shanghai SLAC Laboratory Animal Co. Xenograft models were established by subcutaneous injection of control or gene-manipulated cancer cells into right and left sides, respectively, with 100 μL cell suspension (2.5×10^6^cells for A549 group and 3×10^6^ cells for U87 group). Tumor volumes and body weight were measured every 3 days starting from day 7. The mice were euthanized after 56 days of treatment for A549 and 43 days of treatment for U87. Tumors volume was calculated following the formula: Volume (mm^3^) = (length×width^2^)/2. For subsequent tracer studies, on the final day of the experiment, the tumor-bearing mice received three intravenous injections of 100 μL of 1 M ^13^C-D-glucose (Cambridge Isotope Laboratories, CLM-1396-1) via the tail vein at 15 min intervals prior to sacrifice. One hour later, tumor xenografts were excised, weighed, and flash-frozen at -80°C. All mouse experiments were conducted in accordance with standard operating procedures approved by the Ethical Committee of Longhua Hospital, Shanghai University of Traditional Chinese Medicine: SYXK-Hu-2018-0036.

### Experimental metastasis model

The 4–6 weeks old female thymic BALB/c nude mice (Shanghai SLAC Laboratory Animal Co.,Ltd, Shanghai, China) were used to establish experimental metastasis model. The mice were grouped randomly and injected with either control or gene-manipulated cancer cells (2 × 10^6^cells for the A549 group and the 1.5 × 10^6^ cells for U87 group) via caudal vein. After 50 days of injection for A549 and 60 days for U87, the mice were sacrificed.

### Chemicals and reagents

3-nitrophenylhydrazine (3-NPH)·HCl, 1-ethyl-3-(3-dimethylaminopropyl) carbodiimide (EDC) HCl, and pyridine (HPLC grade), glucose, fructose, glucose 6-phosphate, fructose 6-phosphate, glucose 1-phosphate, fructose 1-phosphate, fructose 1,6-bisphosphate, glyceraldehyde 3-phosphate, dihydroxyacetone phosphate, 2-phosphoglycerate, pyruvate, 6-phosphogluconate, ribulose 5-phosphate, ribose 5-phosphate, sedoheptulose 7-phosphate, erythrose 4-phosphate, citrate, isocitrate, succinate, fumarate, malate, lactate, α-ketoglutarate, oxaloacetate, serine, glutamate, glutamine, aspartate, and hydropyruvate were all purchased from Sigma-Aldrich (St Louis, MO). Optima LC-MS grade water, methanol (MeOH), isopropanol, formic acid, and acetonitrile (ACN) were purchased from Thermo Fisher Scientific. ^13^C-D-glucose (labeled at all six carbons) and ^13^C-D-fructose (labeled at all six carbons) were purchased from Cambridge Isotope Laboratories. FBS: Premium Grade Fetal Bovine Serum (Sigma, F7524-500ml) and Dialyzed Fetal Bovine Serum (gibco, a33820-01). Heat Inactive was performed under 56 °C for 30 min. DMEM, high glucose (gibco, 11965-092). DMEM, no glucose (gibco, 11966-025), Epalrestat (MCE, M08-HY-66009). Amino acids: L-Arginine, Hydrochloride (Millipore Sigma, 181003), Histidine hydrochloride (Millipore Sigma, H0755000), L-Lysine (Millipore Sigma, 62840), L-Phenylalanine (Millipore Sigma, P5482), and L-Valine (Millipore Sigma, V0513). PBS (gibco, 14190-144).

### Targeted metabolomics profiling

Targeted metabolomics analysis of cells was conducted using the Q300 Metabolite Assay Kit from Metabo-Profile Biotechnology of China, based on a method previously published with modifications [33]. Briefly, cell samples were collected on ice and lyophilized. To extract metabolites from lyophilized cells, 150 μL of pre-cooled MeOH: H_2_O (80:20, vol/vol) was added and then lysed by ultrasonication. The lysate was centrifugated at 13,000 rpm at 4°C for 15 min. Afterward, each 25 μL of the supernatant specimen was added to a 96-well plate on a Biomek 4000 workstation (Biomek 4000, Beckman Coulter, Inc., Brea, California, USA). Pre-cooled MeOH with partial internal standards was automatically added to each sample and the mixture was vortexed for 5 min before being centrifuged at 4000 × *g* for 30 min. Next, 30 μL of the supernatant was derivatized with 20 μL of freshly prepared derivative reagents on a new 96-well plate. Following 60 min of derivatization at 30 °C, 350 μL of ice-cold 50% MeOH solution was added to dilute the sample. Subsequently, the reaction mixture was incubated at –20 °C for 20 min to stop the reaction. After the plate was centrifugated at 4000 × *g* at 4 °C for 30 min, an aliquot of 135 μL of supernatant was transferred to another clean 96-well plate containing 15 μL internal standards in each well. Finally, the plate was sealed and 5 μL of the supernatant was injected into the UPLC–MS instrument for analysis.

### Isotope tracing analysis

Isotope tracing of cells and tumor xenografts was performed by Metabo-Profile Biotechnology of China. For the preparation of cell sample: 2 × 10^6^ cells were seeded to a 10 cm dish one day in advance. Cells were washed twice with PBS and then supplemented with glucose free DMEM containing 10% dialyzed FBS supplemented with 25 mM ^13^C_6_-glucose for 24 h before harvesting. Cell pellets were washed three times with pre-cooled PBS, and the lyophilized pellets were stored at −80°C for subsequent analysis. For the preparation of the tumor xenograft sample: the tumor-bearing mice were injected with 100 μL of 1 M ^13^C-glucose via the tail vein three times before sacrifice. One hour later, tumor xenografts were excised, weighed, and flash-frozen at –80 ˚C for future tracer study. Samples were analyzed using the Waters Acquity UPLC system coupled with Xevo-TQs mass spectrometry, based on the previous publications [34]. Metabolites were separated through an Acquity UPLC® BEH C18 column (2.1 × 100 mm, 1.7 μm, Waters) with a VanGuard Pre-Column (2.1 × 5 mm, 1.7 μm, Waters) at 40°C column temperature. An aliquot of 5 μL of the sample was injected into the column and the flow rate remained constant at 0.4 mL/min. The raw data generated by UPLC-MS/MS were analyzed using TargetLynx software version 4.1. Mass distribution vector (MDV) of ^13^C-labeled metabolites, which describes the fractional abundance of each isotopologue normalized to the sum of all possible isotopologues, was measured in all detected metabolites.

### Quantification of ^13^C- labeled metabolites

Standard curves were established for quantitative measurement of metabolites of interest, including ^13^C-glucose, ^13^C-fructose, ^13^C-lactate, and ^13^C-pyruvate. These metabolites were ^13^C labeled at all carbons. After collecting and lyophilizing the cells, they were stored at –80 °C. Metabolites were extracted by cold 80% MeOH (–20 °C) and homogenized for 3 min using a Bullet Blender Tissue Homogenizer (Next Advance, Inc., Averill Park, NY) and centrifugated at 13,500 rpm for 10 min at 4 °C. Derivatization was performed according to the method of Han et al. [35] with minor modifications. An aliquot of 5 μL of supernatant or standard was transferred to a 1.5 mL tube and mixed with 25 μL of 160 mM 3-NPH solution, as well as 25 μL of the mixed 40 mM of EDC/pyridine methanolic solution. Derivatization was conducted by incubating the samples at 30 °C for one hour, followed by 20 min of incubation at –20 °C. Subsequently, supernatant was acquired by centrifugation at 13,000 rpm at 4 °C for 15 min. An aliquot of 5 μL of the derivatives were analyzed via Ultra-high performance liquid chromatography-triple quadrupole mass spectrometry using Waters Xevo TQs or Agilent 6460. The column temperature was maintained at 40 °C. The mobile phase consisting of water with 0.1% formic acid in water (solvent A) and acetonitrile/isopropanol=7/3 (solvent B) was pumped at a flow rate of 0.4 mL/ minutes. The gradient elution program was as follows: 0–8 min, 1–20% B; 8–16 min, 20–98% B; 16.0–16.1 min, 98-1% B; 16.1–18.0 min, 1% B for equilibration of the column. The mass spectrometer was used in negative ESI mode for data acquisition using UPLC/MS. Typical source conditions were as follows: capillary voltage, 3.0 kV; sample cone, 40 V; source temperature, 150 °C; desolvation temperature 500°C; cone gas flow rate 150 L/h; desolvation gas (N2) flow rate 1000 L/h.

Extended Data Table 1 showed the identification of metabolites in cells using UPLC- Acquity Xevo TQs.

### Statistical analysis

Data were presented as the mean ± SEM of at least three independent experiments. Statistical analysis was performed using GraphPad Prism 9. Unless otherwise noted, mean differences between the two groups were evaluated by an unpaired two-tailed *t* test. P < 0.05 was considered statistically significant.

## Supplementary information

supplementary information

supplementary information-original western blots

## Data Availability

All datasets are available from the corresponding author on reasonable request.
