# Supplementary material for: AKR1B1-dependent fructose metabolism enhances malignancy of cancer cells
Source: Cell Death Differ. 2024 Oct 15;31(12):1611–24. doi: 10.1038/s41418-024-01393-4 (PMC11618507; doi:10.1038/s41418-024-01393-4)
Supplement: Supplementary file 1 — supplementary information [file 41418_2024_1393_MOESM1_ESM.pdf]

Supplementary Information for

AKR1B1-dependent Fructose Metabolism Enhances Malignancy of Cancer Cells

Qing Zhao[1,7], Bing Han[2,7], Lu Wang[3,7], Jia Wu[4], Siliang Wang[4], Zhenxing Ren[1], Shouli Wang[1], Haining Yang[5], Michele Carbone[5], Changsheng Dong[4], Gerry Melino[6*], Wen-Lian Chen[4*], Wei Jia[1,3*]

[1]Center for Translational Medicine and Shanghai Key Laboratory of Diabetes Mellitus, Shanghai Sixth People's Hospital Affiliated to Shanghai Jiao Tong University School of Medicine, Shanghai 200233, China

[2]Mayo Clinic, 4500 San Pablo Rd S, Jacksonville, FL 32224, USA

[3]Department of Pharmacology and Pharmacy, University of Hong Kong, Hong Kong, China

[4]Cancer Institute, Longhua Hospital, Shanghai University of Traditional Chinese Medicine, Shanghai 200032, China

[5]Cancer Biology Program, University of Hawaii Cancer Center, Honolulu, HI, 96813, USA

[6]Department of Experimental Medicine, University of Rome "Tor Vergata", 00133, Rome, Italy

[7]These authors contributed equally

[*]Correspondence: weijia2@hku.hk (W.J.), chenwl8412@shutcm.edu.cn (W.-L.C.), melino@uniroma2.it (G.M.)

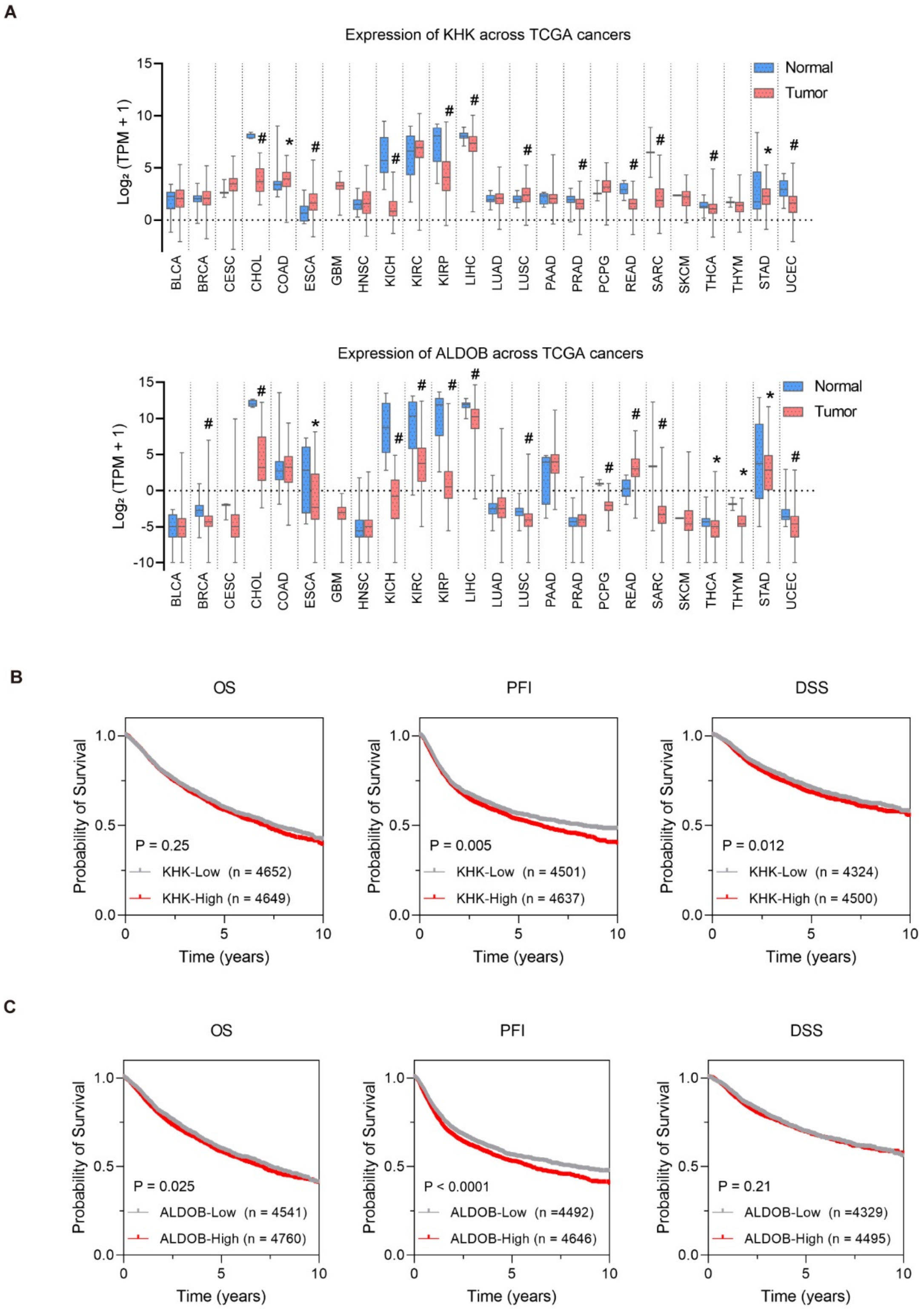
A
Expression of KHK across TCGA cancers
Log₂ (TPM + 1)
Normal
Tumor
BLCA BRCA CESC CHOL COAD ESCA GBM HNSC KICH KIRC KIRP LIHC LUAD LUSC PAAD PRAD PCPG READ SARC SKCM THCA THYM STAD UCEC
Expression of ALDOB across TCGA cancers
Log₂ (TPM + 1)
Normal
Tumor
BLCA BRCA CESC CHOL COAD ESCA GBM HNSC KICH KIRC KIRP LIHC LUAD LUSC PAAD PRAD PCPG READ SARC SKCM THCA THYM STAD UCEC
B
OS
P = 0.25
KHK-Low (n = 4652)
KHK-High (n = 4649)
PFI
P = 0.005
KHK-Low (n = 4501)
KHK-High (n = 4637)
DSS
P = 0.012
KHK-Low (n = 4324)
KHK-High (n = 4500)
Probability of Survival
Time (years)
C
OS
P = 0.025
ALDOB-Low (n = 4541)
ALDOB-High (n = 4760)
PFI
P < 0.0001
ALDOB-Low (n =4492)
ALDOB-High (n = 4646)
DSS
P = 0.21
ALDOB-Low (n =4329)
ALDOB-High (n = 4495)
Probability of Survival
Time (years)

Extended Data Fig. 1 Bioinformatics analysis for the clinical relevance of KHK and ALDOB in cancer patients derived from TCGA database.

(A) (Upper panel) *KHK* expression in multiple cancer tissues from the TCGA database. (Lower panel) *ALDOB* expression in multiple cancer tissues from the TCGA database. *: *t*-test $P < 0.05$; #: *t*-test $P < 0.01$.

(B) Kaplan-Meier curves depicting OS, PFI, and DSS of pan-cancer patients from TCGA database classified by low and high *KHK* expression (RNA-seq). OS, overall survival; PFI, progression free interval; DSS, disease specific survival.

(C) Kaplan-Meier curves depicting OS, PFI, and DSS of pan-cancer patients from TCGA database classified by low and high *ALDOB* expression (RNA-seq). OS, overall survival; PFI, progression free interval; DSS, disease specific survival.

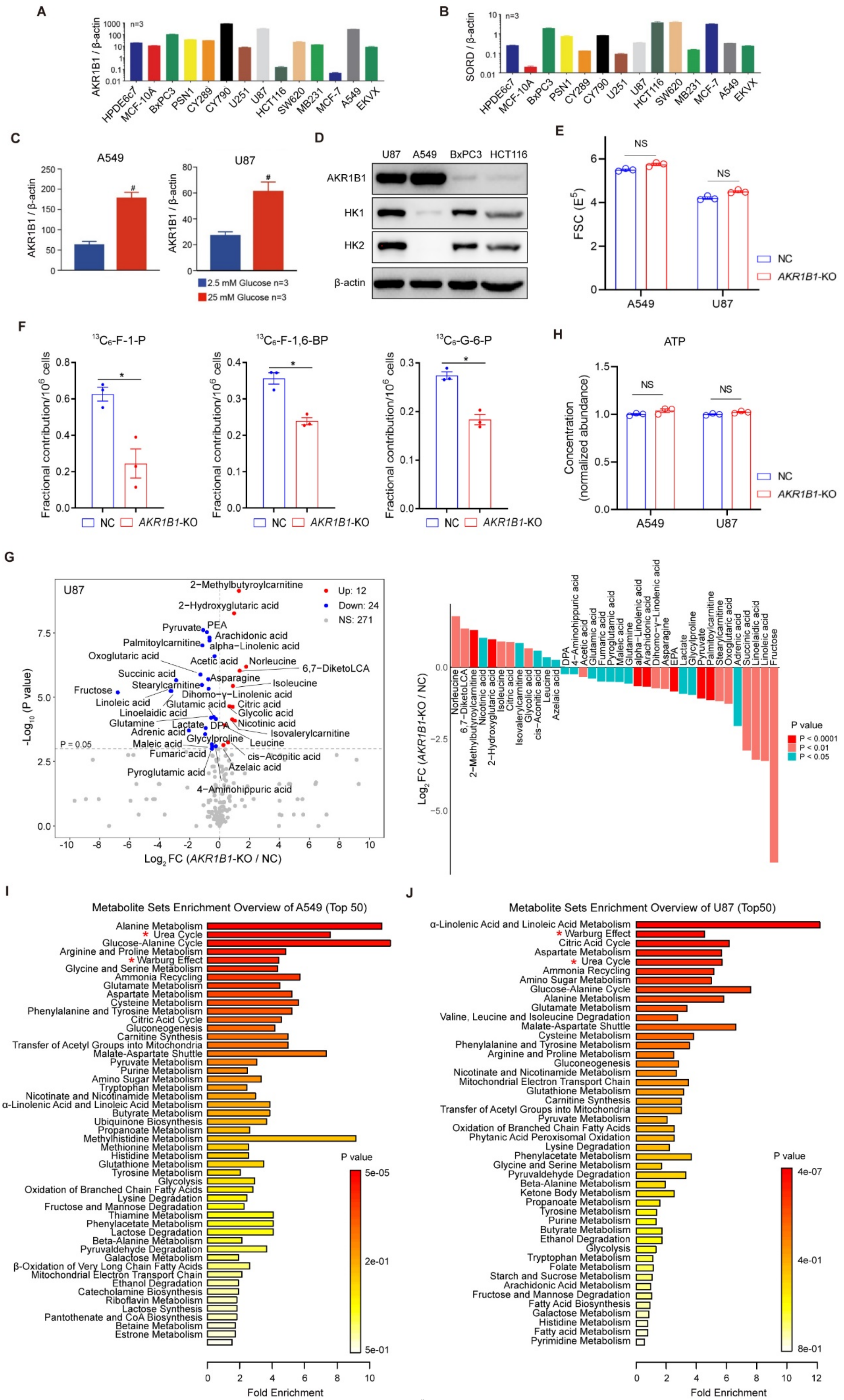
A
AKR1B1 / β-actin
n=3
1000
100
10
1
0.1
0.01
HPDE6c7
MCF-10A
BxPC3
PSN1
CY289
CY790
U251
U87
HCT116
SW620
MB231
MCF-7
A549
EKVX
B
SORD / β-actin
n=3
C
A549
U87
AKR1B1 / β-actin
#
2.5 mM Glucose n=3
25 mM Glucose n=3
D
U87 A549 BxPC3 HCT116
AKR1B1
HK1
HK2
β-actin
E
FSC (E5)
NS
NC
AKR1B1-KO
F
13C6-F-1-P
13C6-F-1,6-BP
13C6-G-6-P
Fractional contribution/10^6 cells
*
NC
AKR1B1-KO
H
ATP
Concentration (normalized abundance)
NS
G
U87
Up: 12
Down: 24
NS: 271
2−Methylbutyroylcarnitine
2−Hydroxyglutaric acid
Pyruvate
PEA
Arachidonic acid
Palmitoylcarnitine
alpha−Linolenic acid
Oxoglutaric acid
Acetic acid
Norleucine
6,7−DiketoLCA
Succinic acid
Asparagine
Stearylcarnitine
Isoleucine
Fructose
Dihomo−γ−Linolenic acid
Linoleic acid
Glutamic acid
Citric acid
Linoelaidic acid
Glycolic acid
Glutamine
Lactate
DPA
Nicotinic acid
Adrenic acid
Isovalerylcarnitine
Maleic acid
Glycylproline
Leucine
Fumaric acid
cis−Aconitic acid
Azelaic acid
Pyroglutamic acid
4−Aminohippuric acid
P = 0.05
-Log10 (P value)
Log2 FC (AKR1B1-KO / NC)
Log2 FC (AKR1B1-KO / NC)
EPA
Pyruvate
Palmitoylcarnitine
Stearylcarnitine
Oxoglutaric acid
Adrenic acid
Succinic acid
Linoelaidic acid
Linoleic acid
Fructose
P value
P < 0.0001
P < 0.01
P < 0.05
I
Metabolite Sets Enrichment Overview of A549 (Top 50)
Alanine Metabolism
* Urea Cycle
Glucose-Alanine Cycle
Arginine and Proline Metabolism
* Warburg Effect
Glycine and Serine Metabolism
Ammonia Recycling
Glutamate Metabolism
Aspartate Metabolism
Cysteine Metabolism
Phenylalanine and Tyrosine Metabolism
Citric Acid Cycle
Gluconeogenesis
Carnitine Synthesis
Transfer of Acetyl Groups into Mitochondria
Malate-Aspartate Shuttle
Pyruvate Metabolism
Purine Metabolism
Amino Sugar Metabolism
Tryptophan Metabolism
Nicotinate and Nicotinamide Metabolism
α-Linolenic Acid and Linoleic Acid Metabolism
Butyrate Metabolism
Ubiquinone Biosynthesis
Propanoate Metabolism
Methylhistidine Metabolism
Methionine Metabolism
Histidine Metabolism
Glutathione Metabolism
Tyrosine Metabolism
Glycolysis
Oxidation of Branched Chain Fatty Acids
Lysine Degradation
Fructose and Mannose Degradation
Thiamine Metabolism
Phenylacetate Metabolism
Lactose Degradation
Beta-Alanine Metabolism
Pyruvaldehyde Degradation
Galactose Metabolism
β-Oxidation of Very Long Chain Fatty Acids
Mitochondrial Electron Transport Chain
Ethanol Degradation
Catecholamine Biosynthesis
Riboflavin Metabolism
Lactose Synthesis
Pantothenate and CoA Biosynthesis
Betaine Metabolism
Estrone Metabolism
P value
5e-05
2e-01
5e-01
Fold Enrichment
J
Metabolite Sets Enrichment Overview of U87 (Top50)
α-Linolenic Acid and Linoleic Acid Metabolism
* Warburg Effect
Citric Acid Cycle
Aspartate Metabolism
* Urea Cycle
Ammonia Recycling
Amino Sugar Metabolism
Glucose-Alanine Cycle
Alanine Metabolism
Glutamate Metabolism
Valine, Leucine and Isoleucine Degradation
Malate-Aspartate Shuttle
Cysteine Metabolism
Phenylalanine and Tyrosine Metabolism
Arginine and Proline Metabolism
Gluconeogenesis
Nicotinate and Nicotinamide Metabolism
Mitochondrial Electron Transport Chain
Glutathione Metabolism
Carnitine Synthesis
Transfer of Acetyl Groups into Mitochondria
Pyruvate Metabolism
Oxidation of Branched Chain Fatty Acids
Phytanic Acid Peroxisomal Oxidation
Lysine Degradation
Phenylacetate Metabolism
Glycine and Serine Metabolism
Pyruvaldehyde Degradation
Beta-Alanine Metabolism
Ketone Body Metabolism
Propanoate Metabolism
Tyrosine Metabolism
Purine Metabolism
Butyrate Metabolism
Ethanol Degradation
Glycolysis
Tryptophan Metabolism
Folate Metabolism
Starch and Sucrose Metabolism
Arachidonic Acid Metabolism
Fructose and Mannose Degradation
Fatty Acid Biosynthesis
Galactose Metabolism
Histidine Metabolism
Fatty acid Metabolism
Pyrimidine Metabolism
P value
4e-07
4e-01
8e-01
Fold Enrichment

34

Extended Data Fig. 2 The expression of enzymes involved in fructose- and glucose-metabolism in multiple cancer cell lines, the ATP concentrations and cell size in nontarget control (NC) and *AKR1B1*-KO cells, and metabolomics analysis of NC and *AKR1B1*-KO U87 cells.

(A-B) Key enzymes of the polyol pathway, *AKR1B1* (A) and *SORD* (B), are widely expressed in multiple cancer cell lines at the mRNA levels (n = 3).

(C) The regulation of AKR1B1 expression by glucose levels in different cancer cells (n = 3).

(D) The expression of AKR1B1, HK1, and HK2 in four different cancer cells.

(E) The cell size in NC as well as *AKR1B1*-KO cells (n = 3).

(F) The amounts of glucose- and fructose-specific metabolites in *AKR1B1*-KO U87 cells. Cells were treated in glucose free DMEM containing 10% dialyzed FBS supplemented with 25 mM ^{13}C-glucose for 24 h and harvested for metabolic flux analysis (n = 3).

(G) The volcano plot showing the perturbed metabolites by *AKR1B1*-KO in U87 cells (Left panel). The bar plot displaying the differential metabolites triggered by *AKR1B1*-KO in U87 cells (Right panel). These metabolites were ranked based on their fold change values and highlighted with different colors according to their P values. FC, fold change.

(H) The ATP concentrations in NC and *AKR1B1*-KO cells (n = 3).

(I-J) Pathway enrichment analysis of differential metabolites was performed using the selected pathway-associated metabolite sets (SMPDB) library.

Data are represented as mean ± SEM. *: *t*-test $P < 0.05$; #: *t*-test $P < 0.01$; NS: not significant.

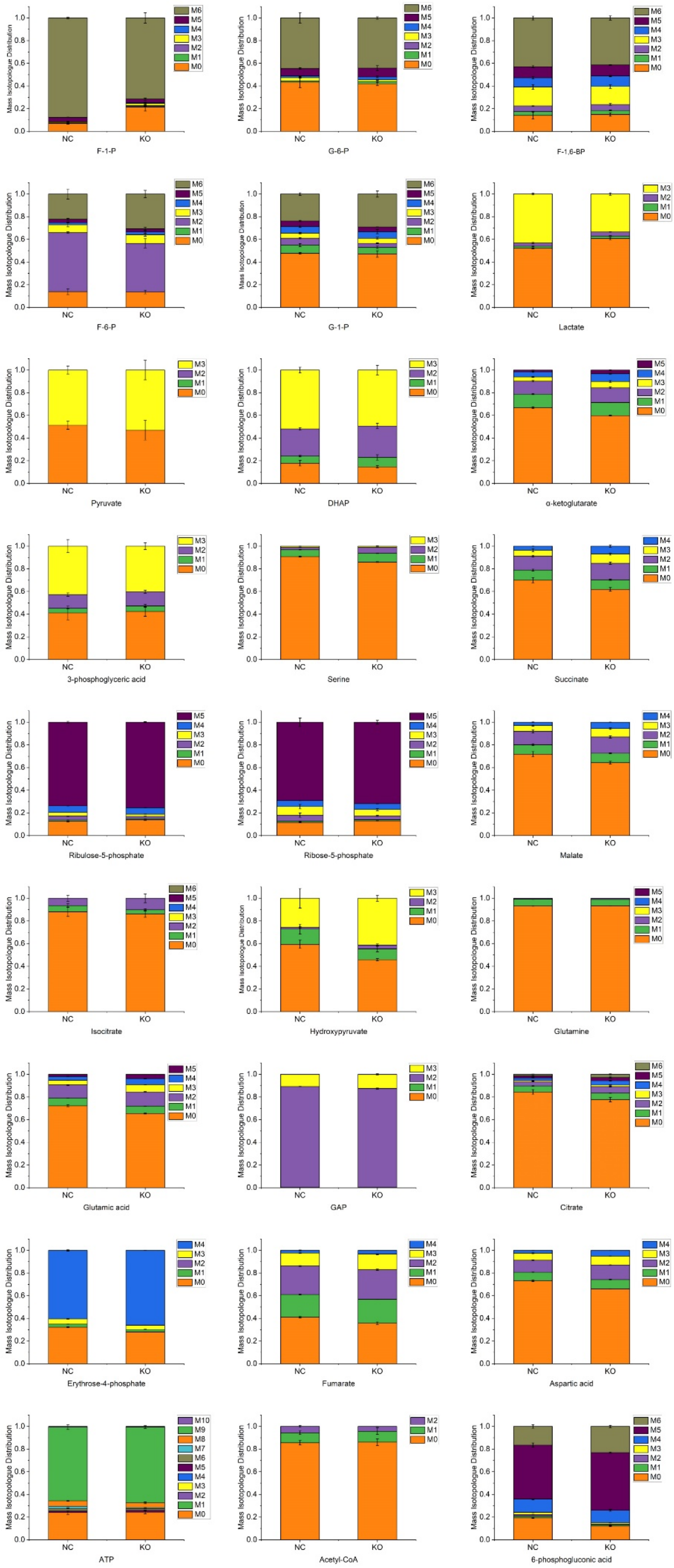
Mass Isotopologue Distribution
NC
KO
M0
M1
M2
M3
M4
M5
M6
M7
M8
M9
M10
F-1-P
G-6-P
F-1,6-BP
F-6-P
G-1-P
Lactate
Pyruvate
DHAP
α-ketoglutarate
3-phosphoglyceric acid
Serine
Succinate
Ribulose-5-phosphate
Ribose-5-phosphate
Malate
Isocitrate
Hydroxypyruvate
Glutamine
Glutamic acid
GAP
Citrate
Erythrose-4-phosphate
Fumarate
Aspartic acid
ATP
Acetyl-CoA
6-phosphogluconic acid

55

Extended Data Fig. 3 Mass isotopologue distributions (MIDs) of metabolites in A549 cells

Mass isotopologue distributions (MIDs) of metabolites in NC and *AKR1B1*-KO A549 cells were measured after 24 h culture with ^{13}C-glucose (labelled at all six carbons). Data are displayed as mean ± SD (n = 3). Glucose-1-phosphate (G-1-P), Fructose-1-phosphate (F-1-P), Fructose-6-phosphate (F-6-P), Glucose-6-phosphate (G-6-P), Fructose-1,6-bisphosphate (F-1,6-BP), Glyceraldehyde-3-phosphate (GAP), Dihydroxyacetone phosphate (DHAP).

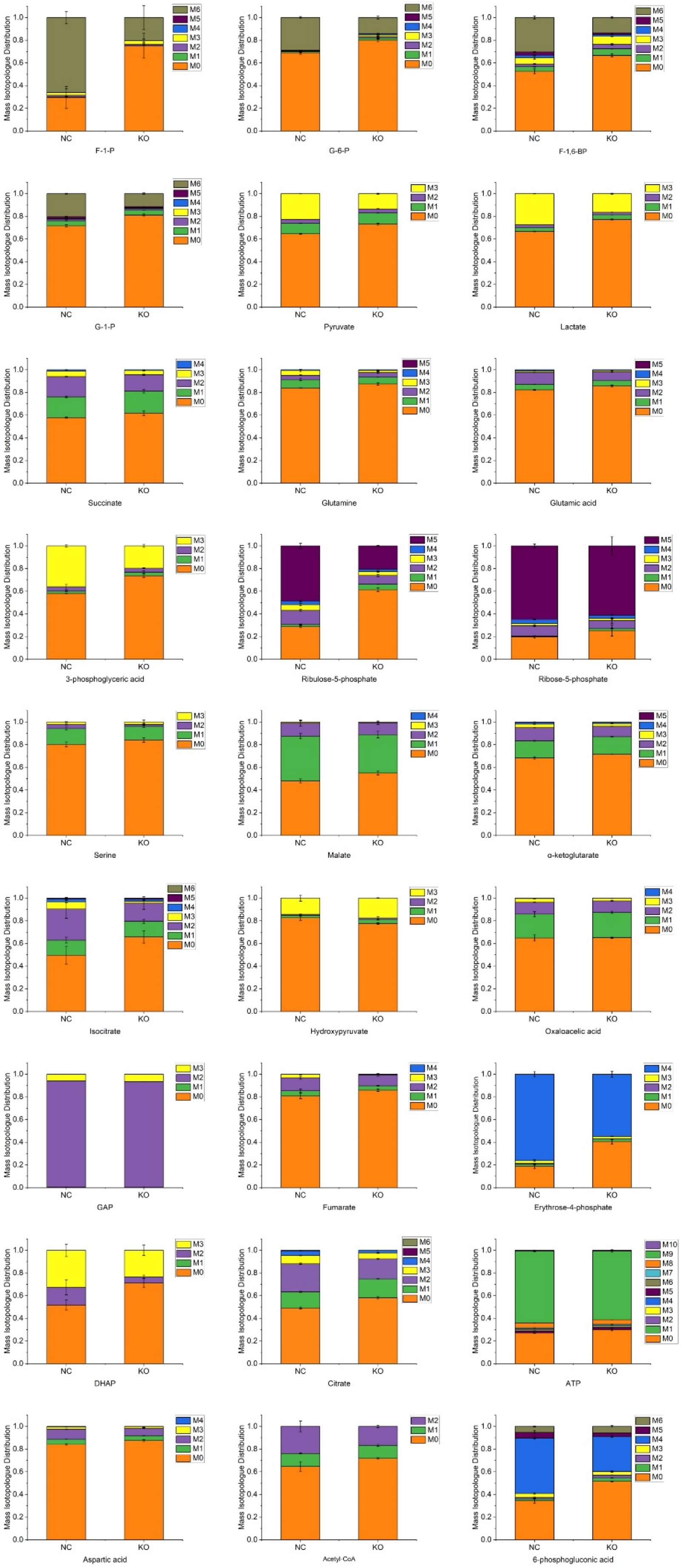
Mass Isotopologue Distribution
NC
KO
M0
M1
M2
M3
M4
M5
M6
M7
M8
M9
M10
F-1-P
G-6-P
F-1,6-BP
G-1-P
Pyruvate
Lactate
Succinate
Glutamine
Glutamic acid
3-phosphoglyceric acid
Ribulose-5-phosphate
Ribose-5-phosphate
Serine
Malate
α-ketoglutarate
Isocitrate
Hydroxypyruvate
Oxaloacelic acid
GAP
Fumarate
Erythrose-4-phosphate
DHAP
Citrate
ATP
Aspartic acid
Acetyl CoA
6-phosphogluconic acid

Extended Data Fig. 4 Mass isotopologue distributions (MIDs) of metabolites in U87 cells

Mass isotopologue distributions (MIDs) of metabolites in NC and *AKR1B1*-KO in U87 cells were measured after 24 h culture with ^{13}C-glucose (labelled at all six carbons). The data are displayed as mean ± SD (n = 3). Glucose 1-phosphate (G-1-P), Fructose 1-phosphate (F-1-P), Fructose 6-phosphate (F-6-P), Glucose 6-phosphate (G-6-P), Fructose 1,6-bisphosphate (F-1,6-BP), D-Glyceraldehyde 3-phosphate (GAP), Dihydroxyacetone phosphate (DHAP).

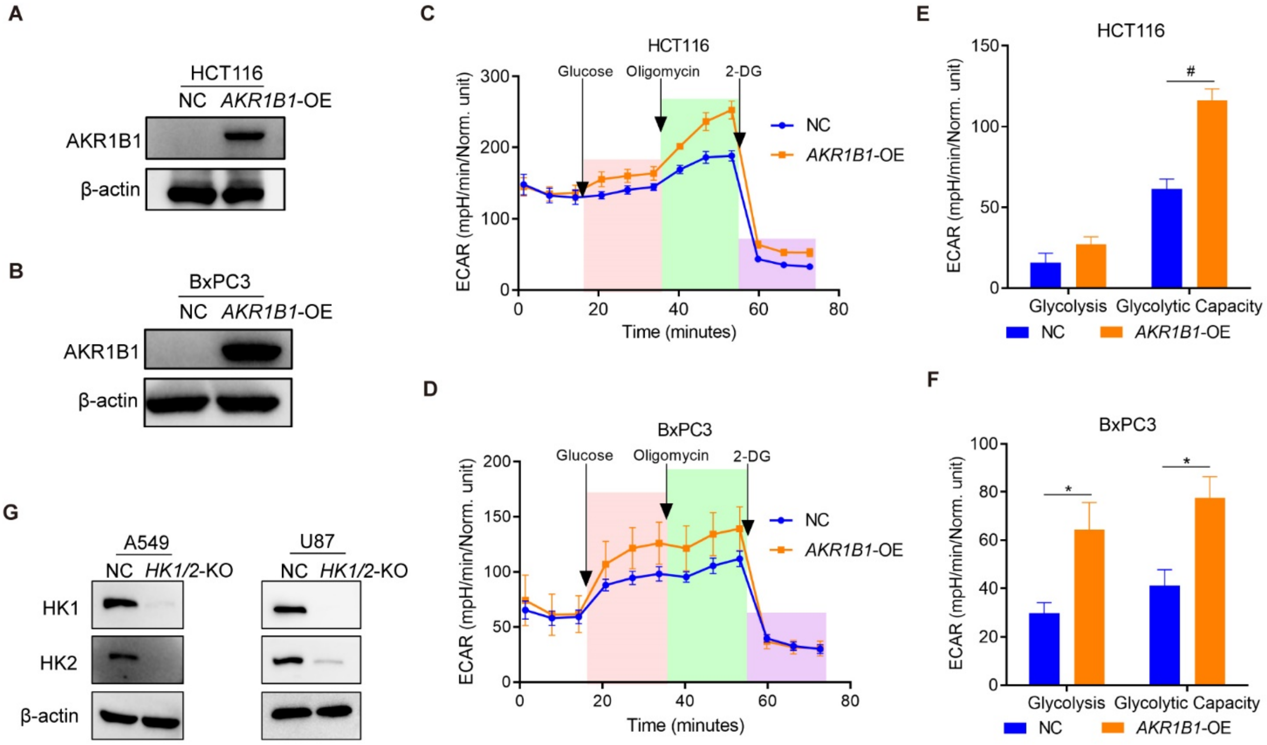
A
HCT116
NC AKR1B1-OE
AKR1B1
β-actin
B
BxPC3
NC AKR1B1-OE
AKR1B1
β-actin
C
HCT116
Glucose Oligomycin 2-DG
ECAR (mpH/min/Norm. unit)
Time (minutes)
NC
AKR1B1-OE
D
BxPC3
Glucose Oligomycin 2-DG
ECAR (mpH/min/Norm. unit)
Time (minutes)
NC
AKR1B1-OE
E
HCT116
ECAR (mpH/min/Norm. unit)
#
Glycolysis Glycolytic Capacity
NC AKR1B1-OE
F
BxPC3
ECAR (mpH/min/Norm. unit)
*
*
Glycolysis Glycolytic Capacity
NC AKR1B1-OE
G
A549
NC HK1/2-KO
U87
NC HK1/2-KO
HK1
HK2
β-actin

Extended Data Fig. 5 The glycolysis stress test in NC and *AKR1B1*-OE cells of HCT116 and BxPC3, and protein levels between NC and *HK1/2*-KO cells from A549 and U87.

(A-B) Homologous recombination technology was used to overexpress *AKR1B1* in cancer cells, resulting in a significant increase in AKR1B1 expression in both HCT116 and BxPC3 cells.

(C-F) The glycolysis stress test revealed the enhanced glycolysis by *AKR1B1*-OE in HCT116 (C, E) and BxPC3 (D, F) ($n = 4$).

(G) The comparison of HK1 and HK2 protein levels between NC and *HK1/2*-KO cells.

Data are represented as mean ± SEM. *: *t*-test $P < 0.05$; #: *t*-test $P < 0.01$.

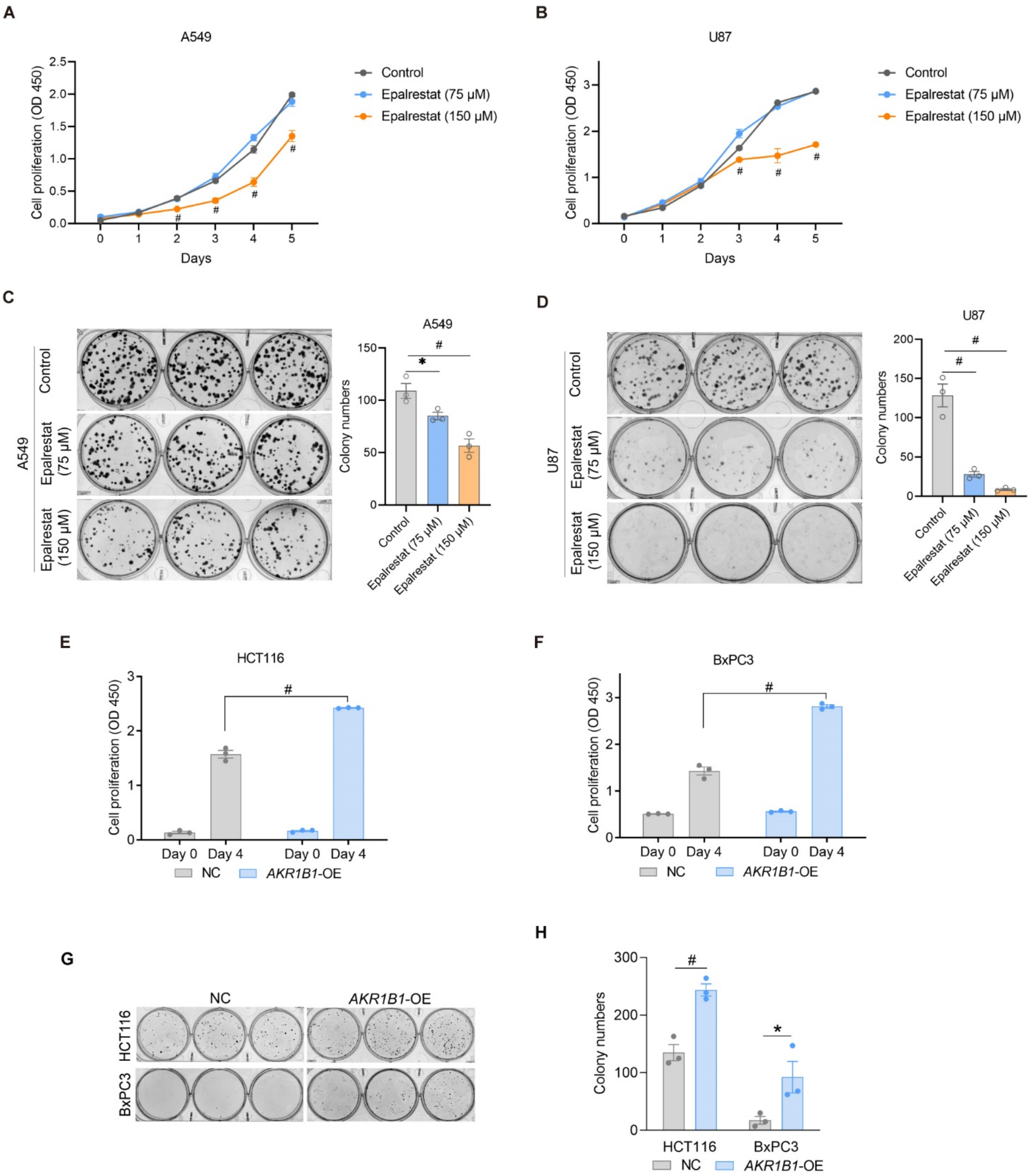
A
A549
Cell proliferation (OD 450)
Days
Control
Epalrestat (75 μM)
Epalrestat (150 μM)
B
U87
Cell proliferation (OD 450)
Days
Control
Epalrestat (75 μM)
Epalrestat (150 μM)
C
A549
Control
Epalrestat (75 μM)
Epalrestat (150 μM)
Colony numbers
D
U87
Control
Epalrestat (75 μM)
Epalrestat (150 μM)
Colony numbers
E
HCT116
Cell proliferation (OD 450)
Day 0
Day 4
NC
AKR1B1-OE
F
BxPC3
Cell proliferation (OD 450)
Day 0
Day 4
NC
AKR1B1-OE
G
NC
AKR1B1-OE
HCT116
BxPC3
H
Colony numbers
HCT116
BxPC3
NC
AKR1B1-OE

Extended Data Fig. 6 The effects of epalrestat and *AKR1B1*-OE on cancer cell proliferation and colony formation.

(A-B) The proliferation of A549 (A) and U87 (B) cells cultured in high glucose DMEM medium containing 10% FBS with or without epalrestat treatment (n = 3).

(C-D) The colony formation of A549 (C) and U87 (D) cells cultured in high glucose DMEM medium containing 20% FBS with or without epalrestat treatment (n = 3).

(E-F) Overexpression of *AKR1B1* accelerated cell proliferation of HCT116 and BxPC3 cells cultured in high glucose DMEM medium containing 10% FBS *in vitro* (n = 3).

(G-H) *AKR1B1*-OE boosted the colony formation of HCT116 and BxPC3 cells *in vitro* (n = 3). Cells were cultured in high glucose DMEM medium containing 20% FBS for 14 days.

Data are represented as mean ± SEM. *: *t*-test $P < 0.05$; #: *t*-test $P < 0.01$.

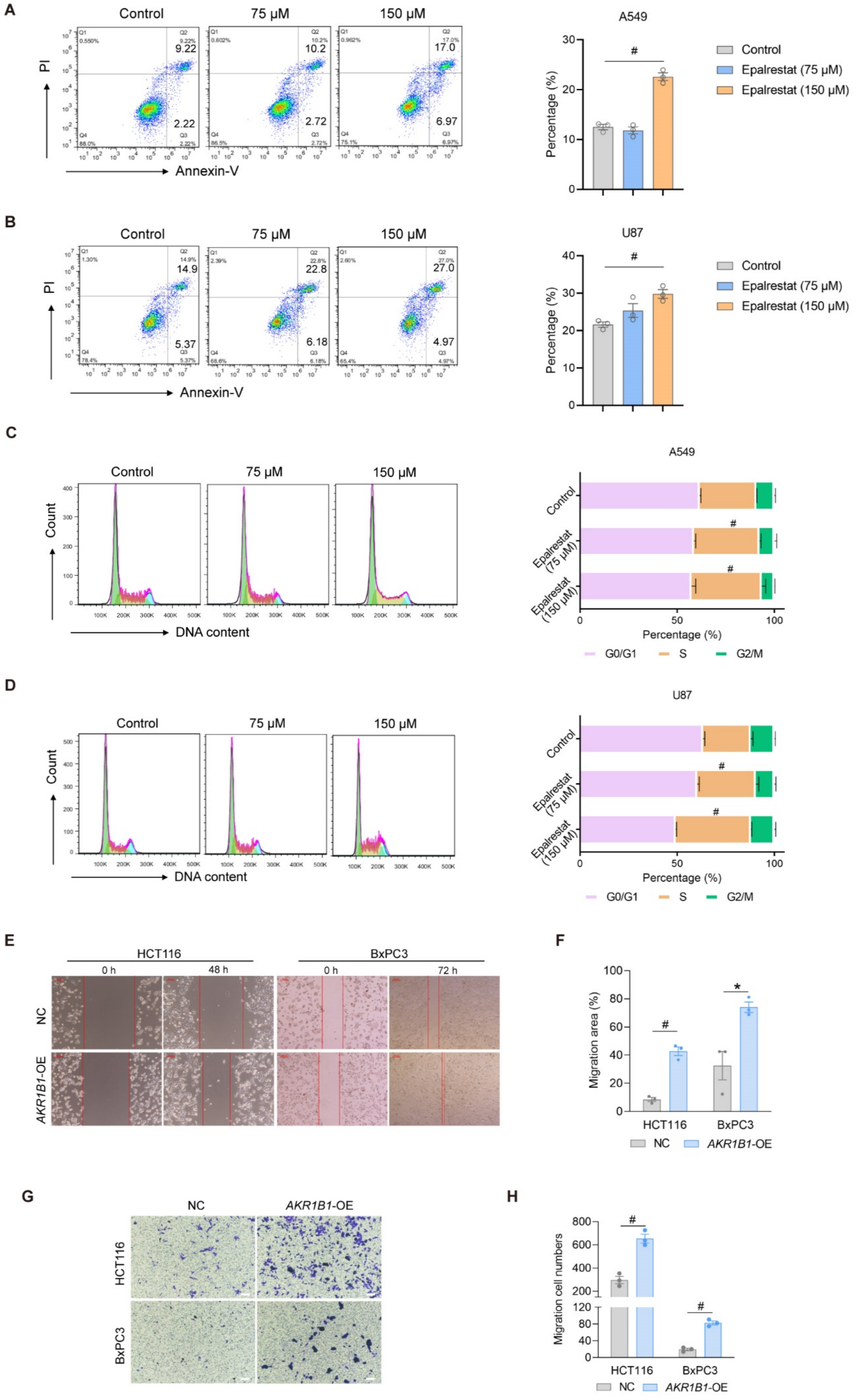
A
Control
75 μM
150 μM
PI
Annexin-V
9.22
2.22
10.2
2.72
17.0
6.97
A549
Percentage (%)
#
Control
Epalrestat (75 μM)
Epalrestat (150 μM)
B
14.9
5.37
22.8
6.18
27.0
4.97
U87
C
Count
DNA content
G0/G1
S
G2/M
D
E
HCT116
BxPC3
0 h
48 h
72 h
NC
AKR1B1-OE
F
Migration area (%)
*
G
H
Migration cell numbers

Extended Data Fig. 7 The effects of epalrestat on cell apoptosis and cell cycle progression in A549 and U87 cells, and the influence of *AKR1B1*-OE on cell migration of HCT116 and BxPC3 cells.

(A-B) Flow cytometry was used to detect cell apoptosis of A549 (A) and U87 (B) cells cultured in high glucose DMEM medium containing 10% FBS with or without epalrestat treatment (n = 3).

(C-D) Flow cytometry was used to examined cell cycle of A549 (C) and U87 (D) cells, which were cultured in high glucose DMEM medium containing 10% FBS with or without epalrestat treatment (n = 3).

(E-F) *AKR1B1*-OE promoting cell migration of HCT116 and BxPC3 cells in wound healing assay. NC and *AKR1B1*-OE cells were cultured in high glucose DMEM medium containing 2% FBS for wound healing assay (n = 3). Bar plot on the right side showing the quantitative and statistical results.

(G-H) *AKR1B1*-OE fostering cell migration of HCT116 and BxPC3 cells in transwell assay. NC and *AKR1B1*-OE cells were seeded in the upper chambers in high glucose DMEM medium, and the lower chamber was the same medium containing 10% FBS (n = 3). Bar plot on the right side showing the quantitative and statistical results. Scale bar is 100 μm.

Data are represented as mean ± SEM. *: *t*-test $P < 0.05$; #: *t*-test $P < 0.01$.

Extended Data Table 1.

Quantification of metabolites using Waters Acquity Xevo TQs

Compound	Parent ion	Daughter ion	Cone（V）	CE（V）	RT (min)
$^{13}C_6$-glucose	319.9	209	25	10	3.21
$^{13}C_6$-fructose	320.0957	240.082	40	10	3.67
$^{13}C_3$-lactate	227	136.7	30	20	3.22
$^{13}C_3$-pyruvic acid	360.6	136.8	46	25	8.22
D_7-glucose	321	209.0913	48	10	3.19
