# Supplementary material for: AKR1B1-dependent fructose metabolism enhances malignancy of cancer cells
Source: Cell Death Differ. 2024 Oct 15;31(12):1611–24. doi: 10.1038/s41418-024-01393-4 (PMC11618507; doi:10.1038/s41418-024-01393-4)
Supplement: Supplementary file 2 — supplementary information-original western blots [file 41418_2024_1393_MOESM2_ESM.pdf]

Fig. 1C

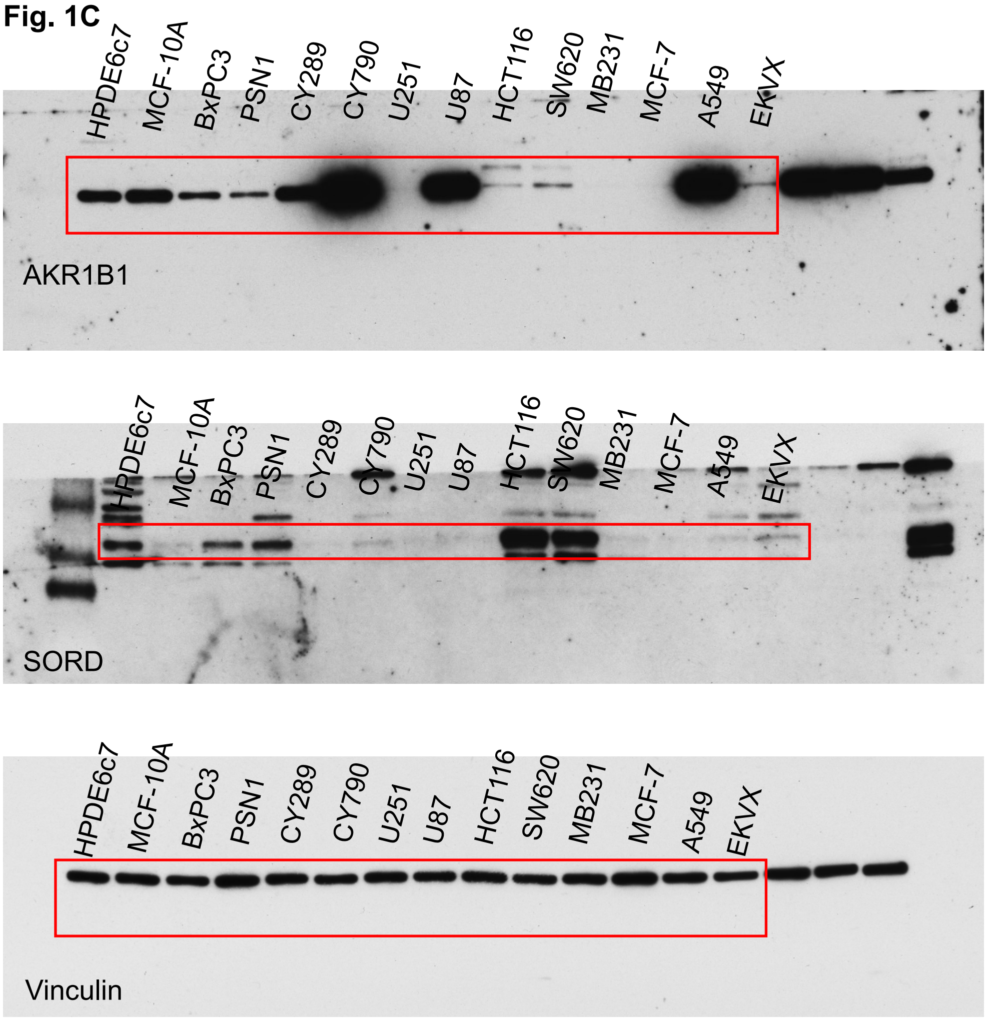
HPDE6c7
MCF-10A
BxPC3
PSN1
CY289
CY790
U251
U87
HCT116
SW620
MB231
MCF-7
A549
EKVX
AKR1B1
SORD
Vinculin

Fig. 2A

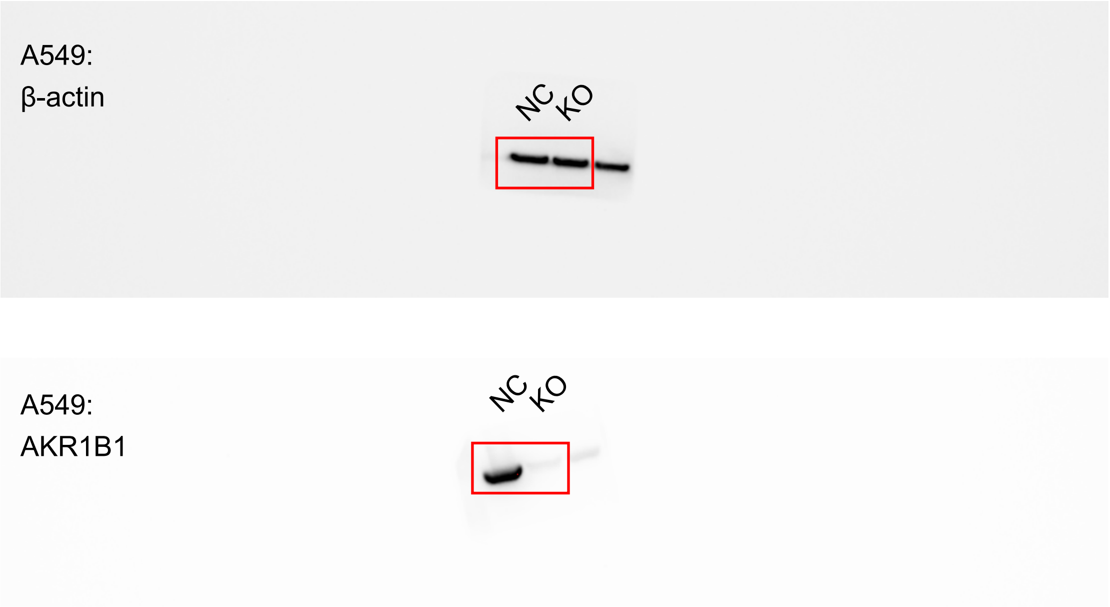
A549:
β-actin
NC
KO
A549:
AKR1B1
NC
KO

U87:
β-actin

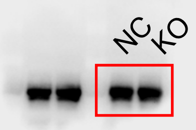

U87:
AKR1B1

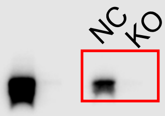

Fig. 4E

A549:
β-actin

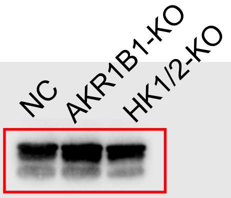

A549:
AKR1B1

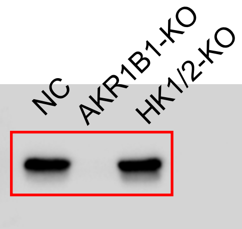

U87:
β-actin

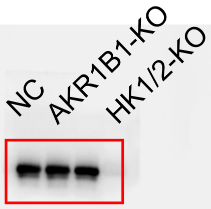

U87:
AKR1B1

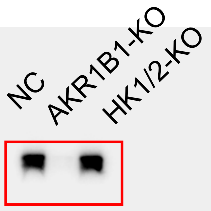

Fig. 6K

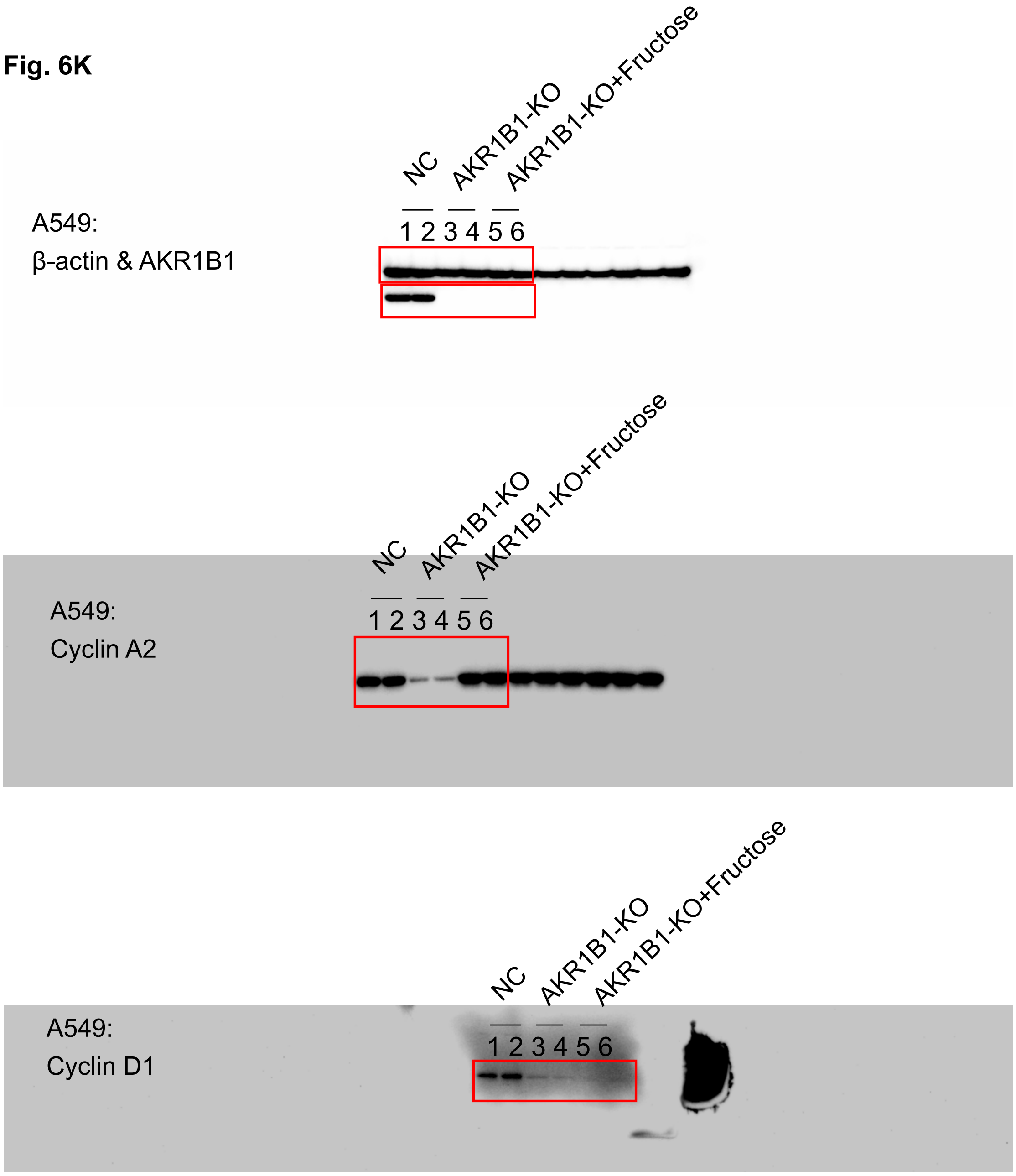

NC
AKR1B1-KO
AKR1B1-KO+Fructose
1 2 3 4 5 6
A549:
β-actin & AKR1B1
NC
AKR1B1-KO
AKR1B1-KO+Fructose
1 2 3 4 5 6
A549:
Cyclin A2
NC
AKR1B1-KO
AKR1B1-KO+Fructose
1 2 3 4 5 6
A549:
Cyclin D1

Fig. 6L

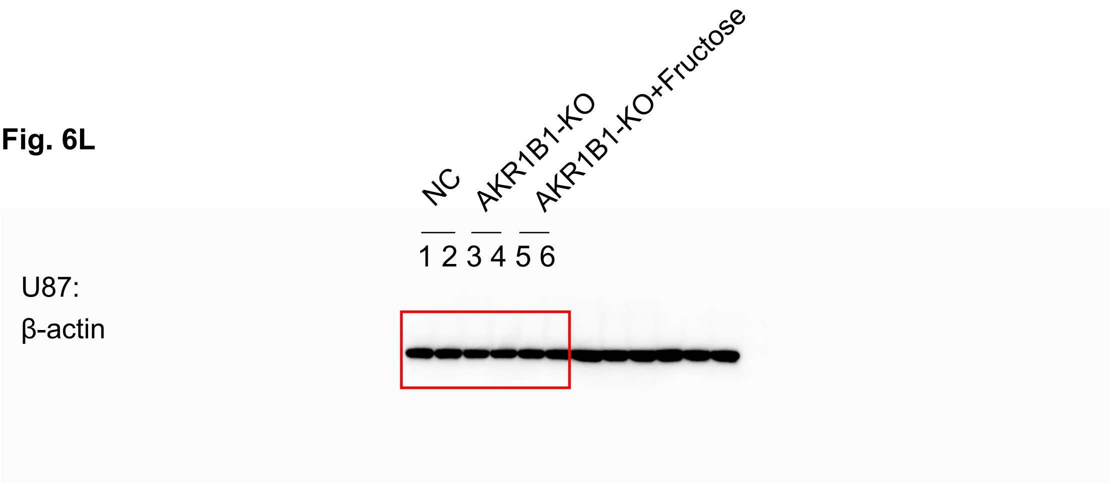

NC
AKR1B1-KO
AKR1B1-KO+Fructose
1 2 3 4 5 6
U87:
β-actin

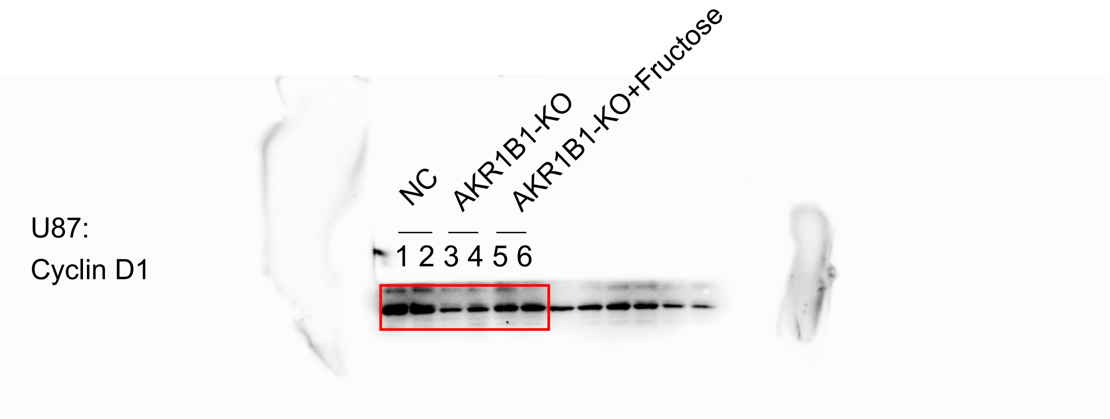
U87:
Cyclin D1
NC
AKR1B1-KO
AKR1B1-KO+Fructose
1 2 3 4 5 6

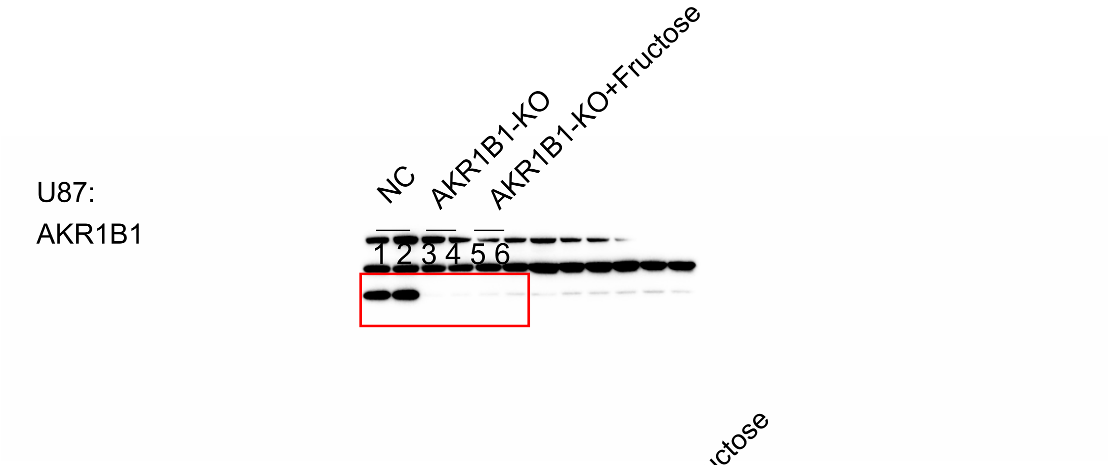
U87:
AKR1B1
NC
AKR1B1-KO
AKR1B1-KO+Fructose
1 2 3 4 5 6

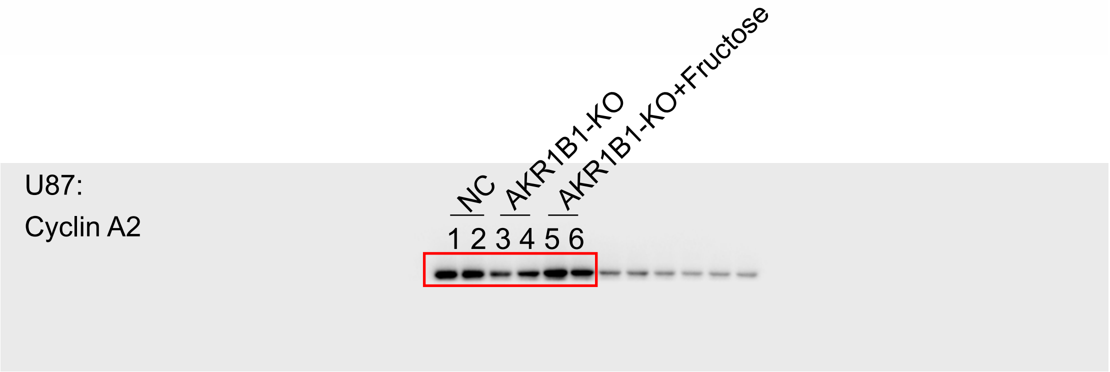
U87:
Cyclin A2
NC
AKR1B1-KO
AKR1B1-KO+Fructose
1 2 3 4 5 6

Fig7. L

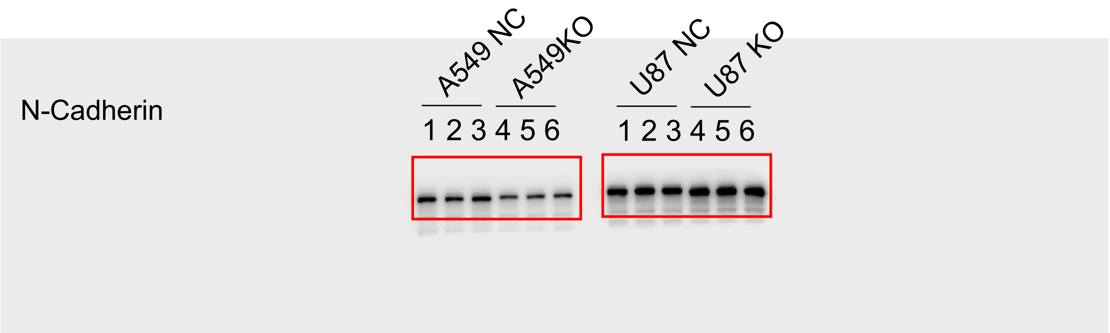
N-Cadherin
A549 NC
A549KO
U87 NC
U87 KO
1 2 3 4 5 6
1 2 3 4 5 6

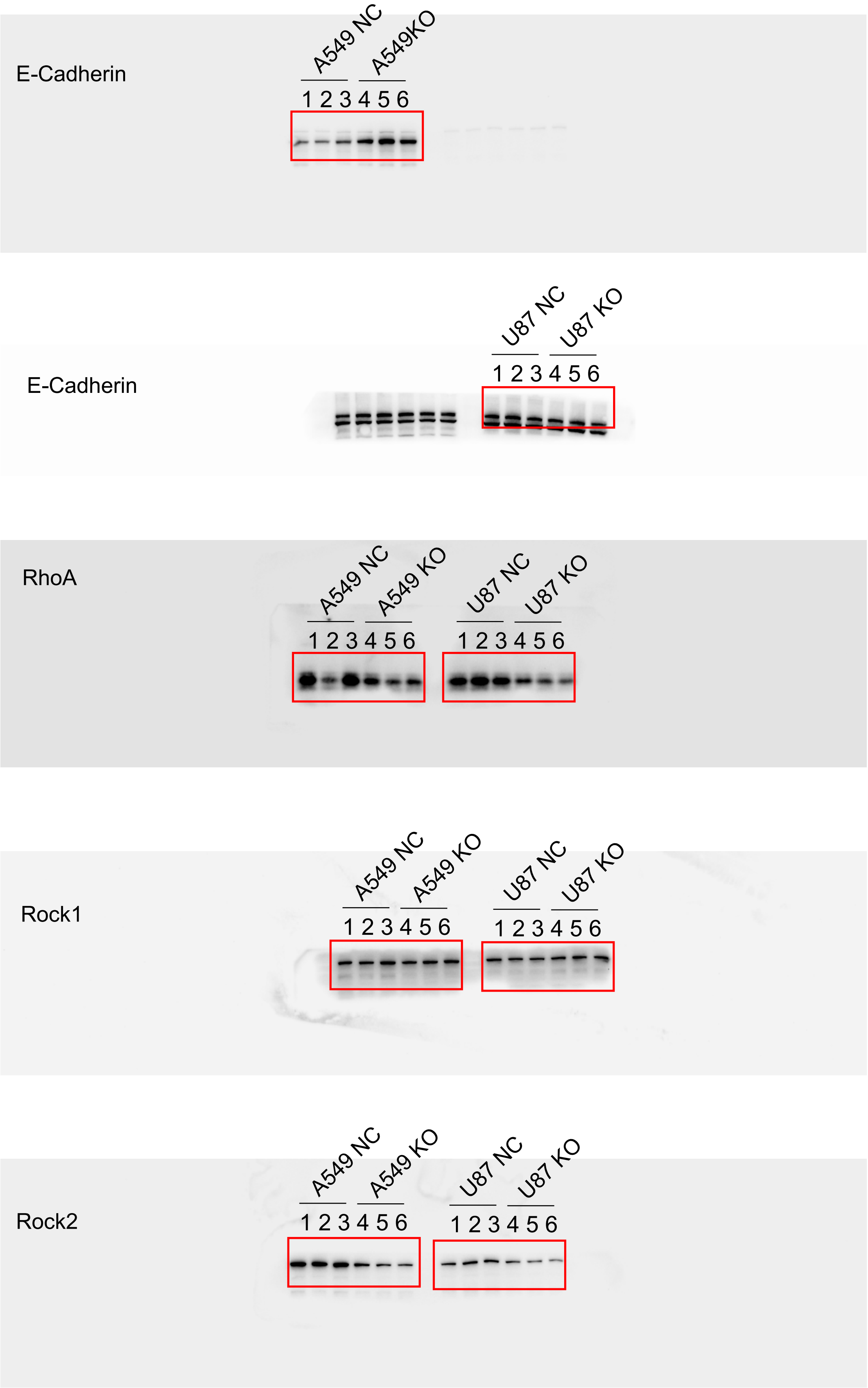
E-Cadherin
A549 NC
A549KO
1 2 3 4 5 6
E-Cadherin
U87 NC
U87 KO
1 2 3 4 5 6
RhoA
A549 NC
A549 KO
1 2 3 4 5 6
U87 NC
U87 KO
1 2 3 4 5 6
Rock1
A549 NC
A549 KO
1 2 3 4 5 6
U87 NC
U87 KO
1 2 3 4 5 6
Rock2
A549 NC
A549 KO
1 2 3 4 5 6
U87 NC
U87 KO
1 2 3 4 5 6

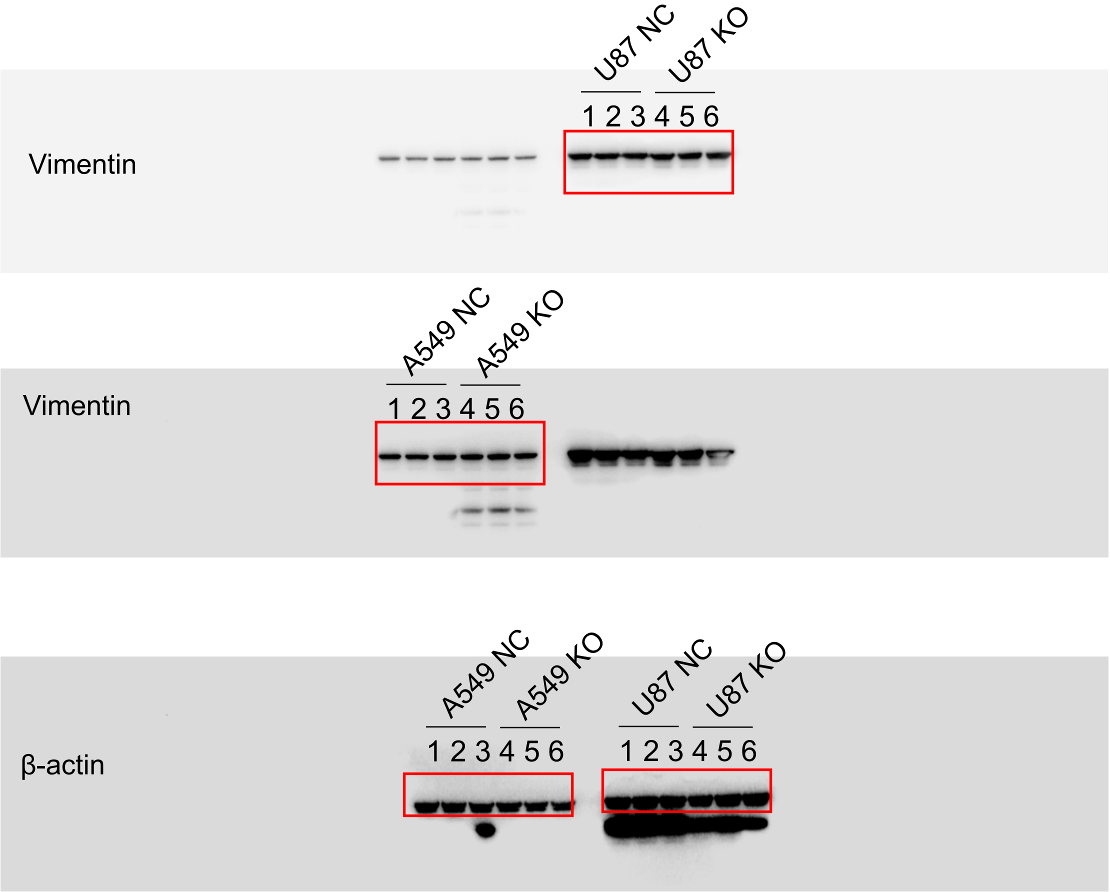
U87 NC
U87 KO
1 2 3 4 5 6
Vimentin
A549 NC
A549 KO
1 2 3 4 5 6
Vimentin
A549 NC
A549 KO
U87 NC
U87 KO
1 2 3 4 5 6
1 2 3 4 5 6
β-actin

Fig. 7M

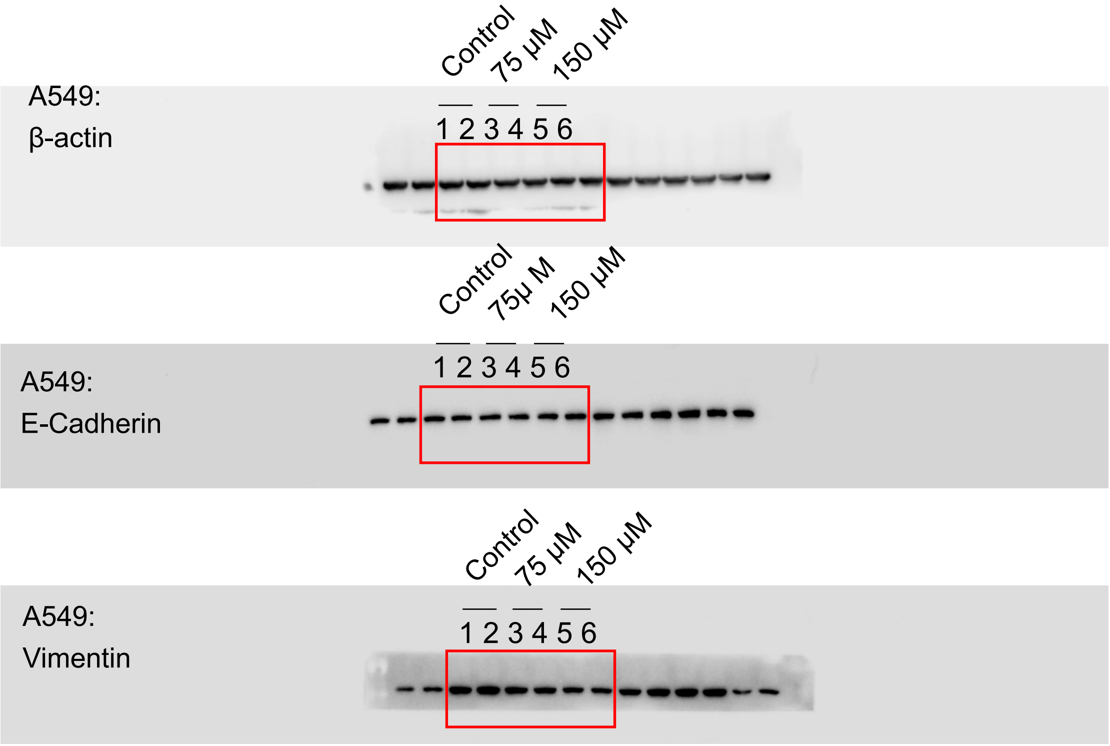
Control
75 μM
150 μM
1 2 3 4 5 6
A549:
β-actin
Control
75μ M
150 μM
1 2 3 4 5 6
A549:
E-Cadherin
Control
75 μM
150 μM
1 2 3 4 5 6
A549:
Vimentin

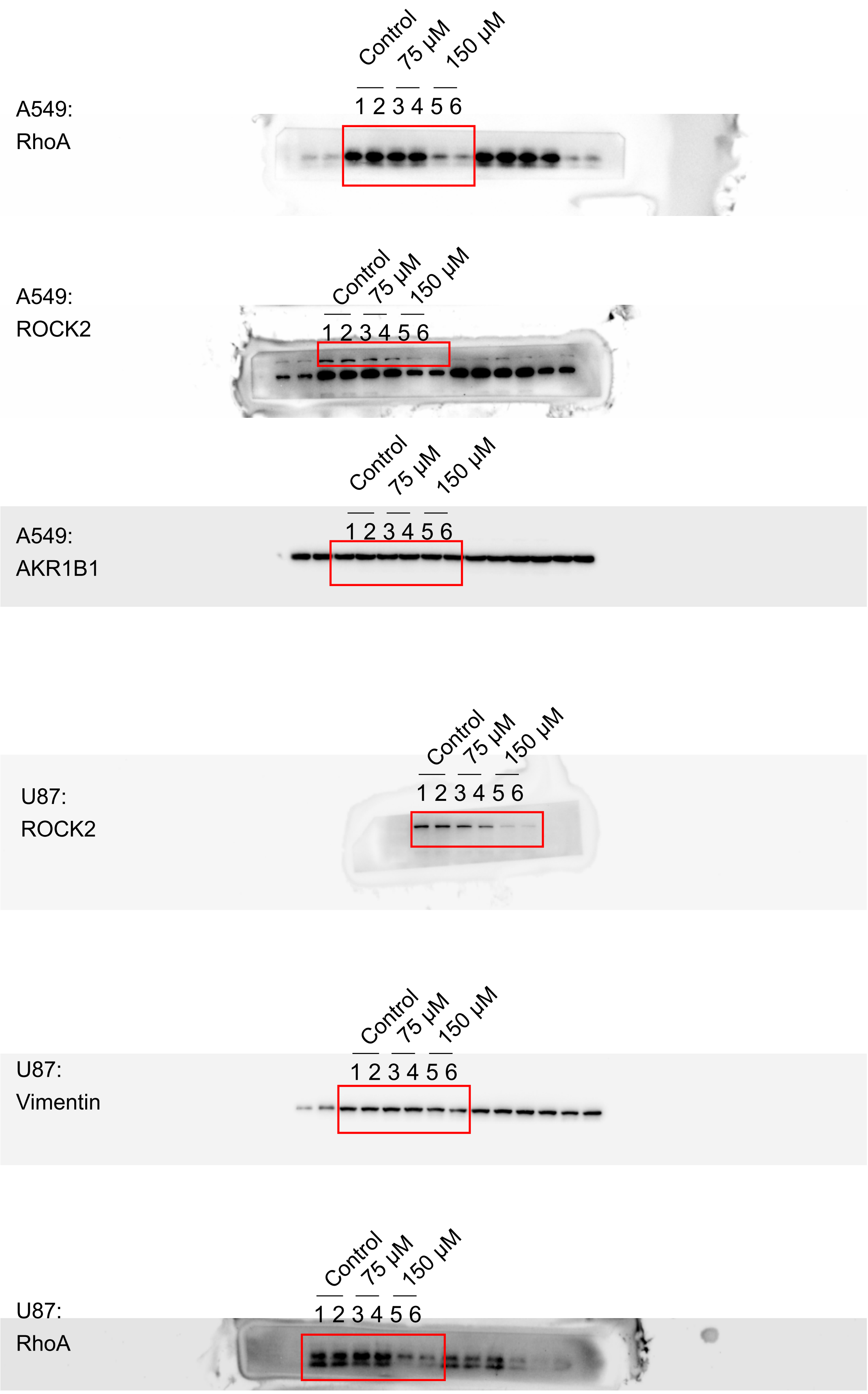

A549:
RhoA
Control
75 μM
150 μM
1 2 3 4 5 6
A549:
ROCK2
Control
75 μM
150 μM
1 2 3 4 5 6
A549:
AKR1B1
Control
75 μM
150 μM
1 2 3 4 5 6
U87:
ROCK2
Control
75 μM
150 μM
1 2 3 4 5 6
U87:
Vimentin
Control
75 μM
150 μM
1 2 3 4 5 6
U87:
RhoA
Control
75 μM
150 μM
1 2 3 4 5 6

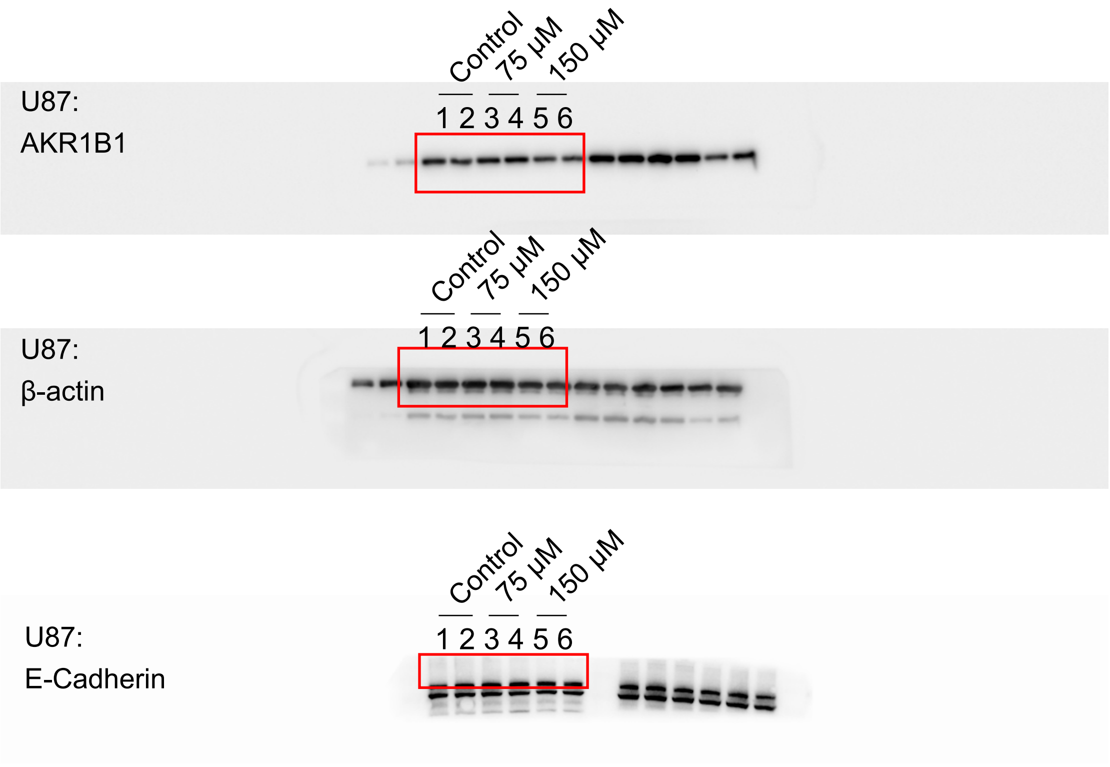
U87:
AKR1B1
Control
75 μM
150 μM
1 2 3 4 5 6
U87:
β-actin
Control
75 μM
150 μM
1 2 3 4 5 6
U87:
E-Cadherin
Control
75 μM
150 μM
1 2 3 4 5 6

Fig. 7N

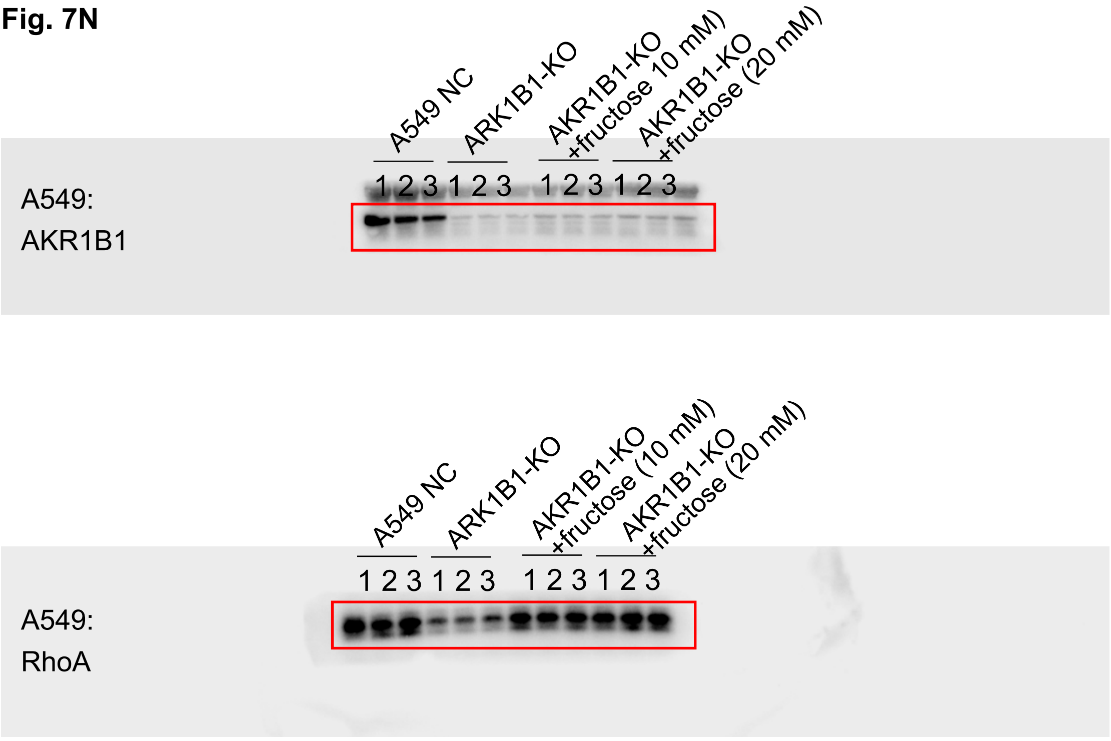
A549:
AKR1B1
A549 NC
ARK1B1-KO
AKR1B1-KO
+fructose 10 mM)
AKR1B1-KO
+fructose (20 mM)
1 2 3 1 2 3 1 2 3 1 2 3
A549:
RhoA
A549 NC
ARK1B1-KO
AKR1B1-KO
+fructose (10 mM)
AKR1B1-KO
+fructose (20 mM)
1 2 3 1 2 3 1 2 3 1 2 3

A549:
β-actin

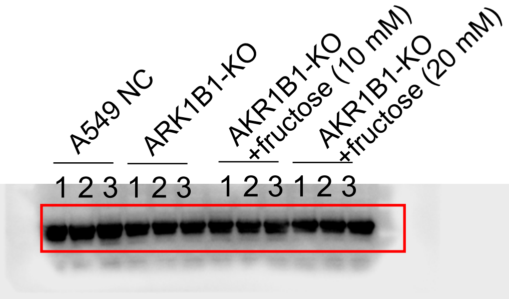

A549:
Rock2

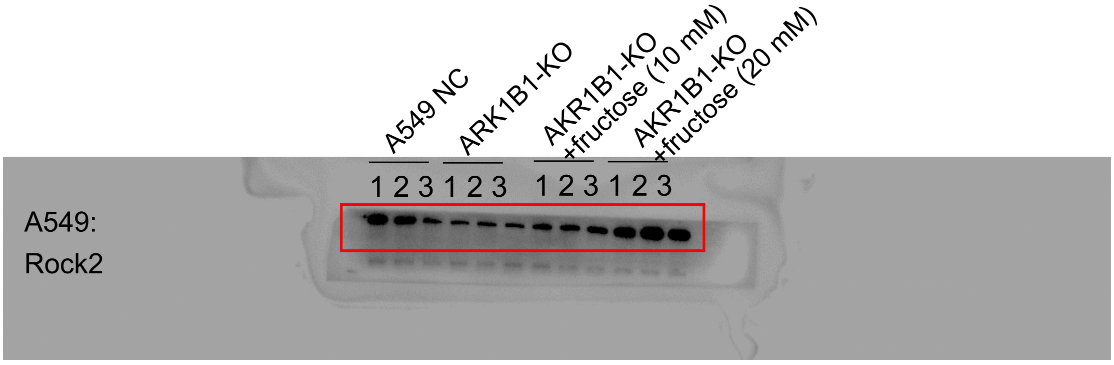

U87:
Rock2

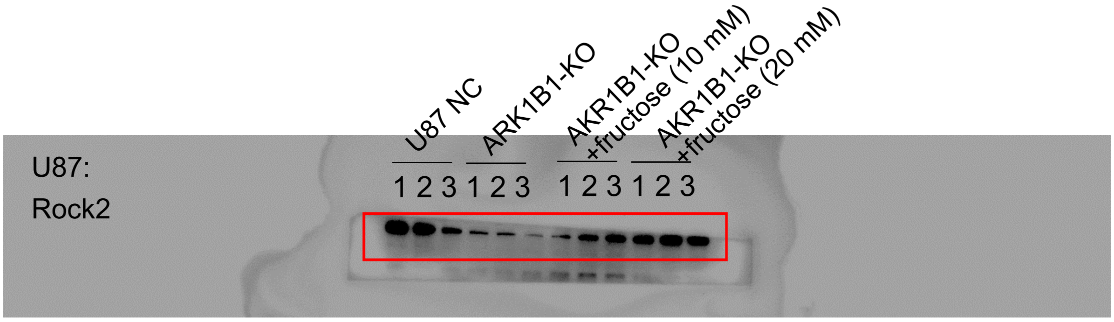

U87:
AKR1B1

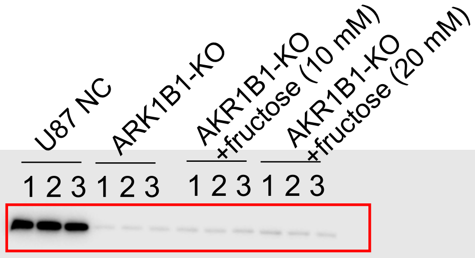

U87:
β-actin

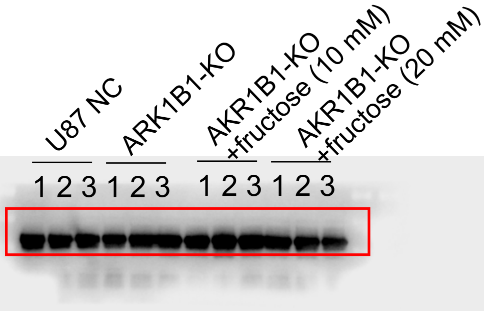

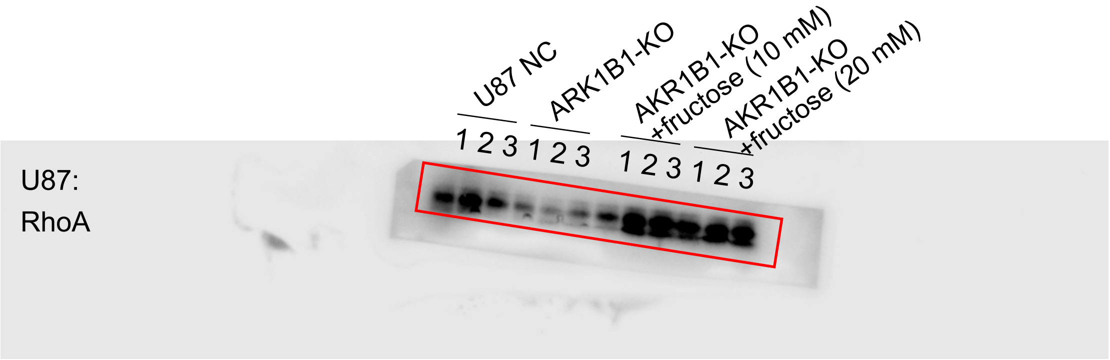

Extended Data Fig. 2D

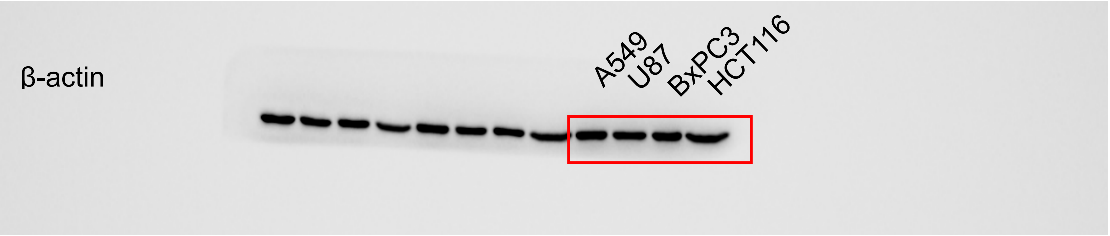

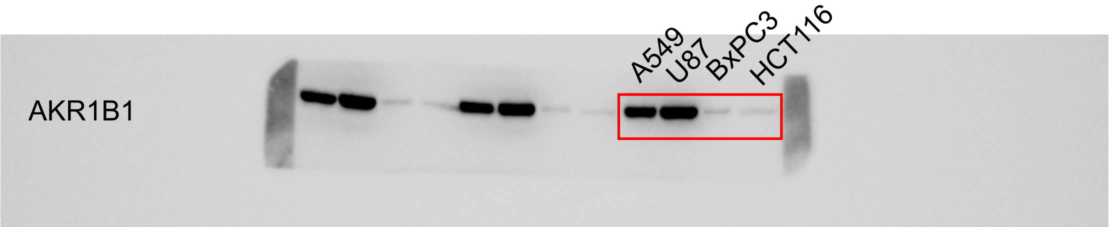

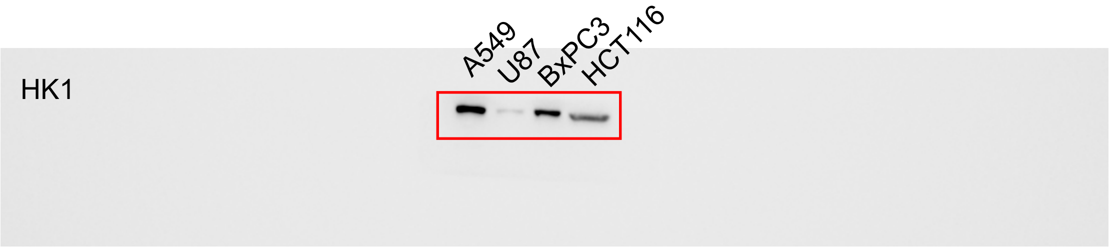

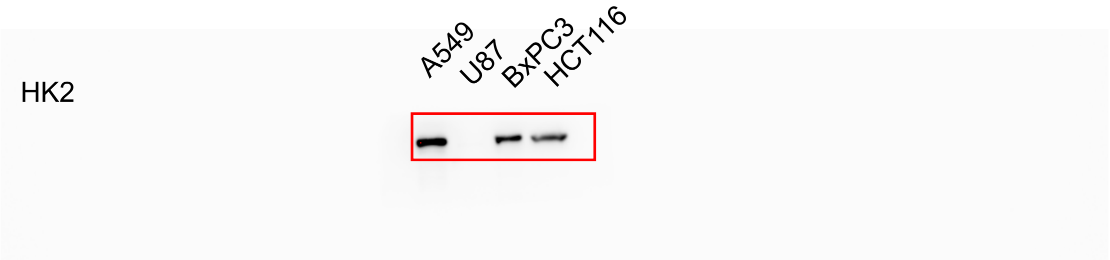

Extended Data Fig. 5A

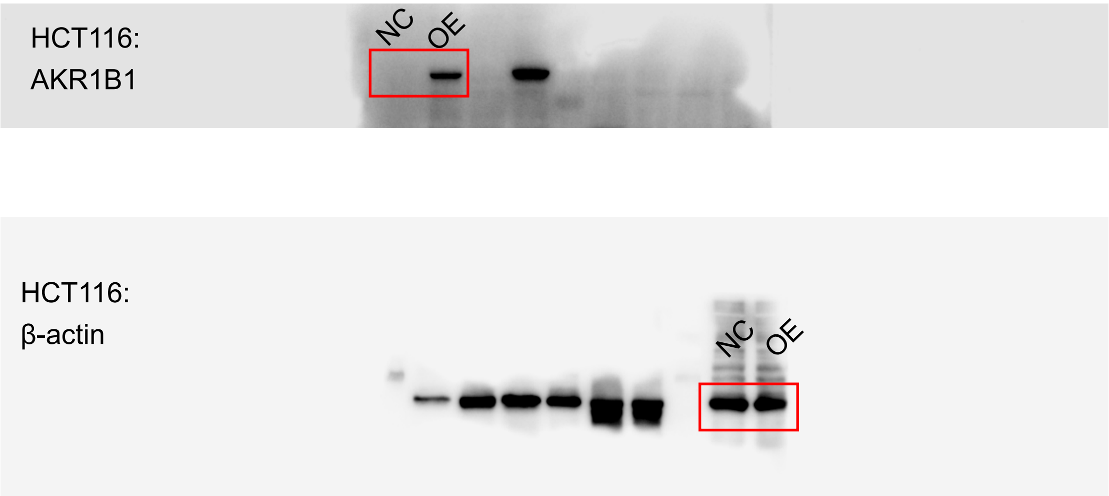

Extended Data Fig. 5B

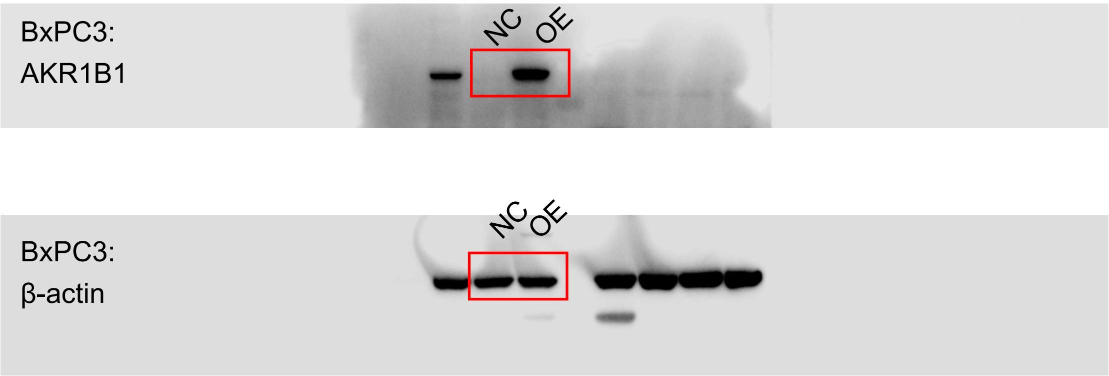

Extended Data Fig. 5G

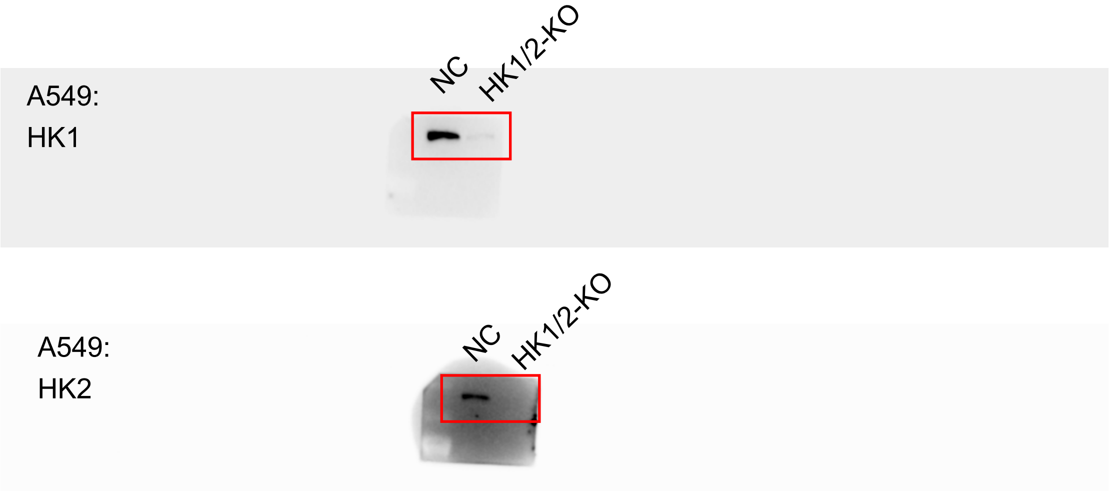

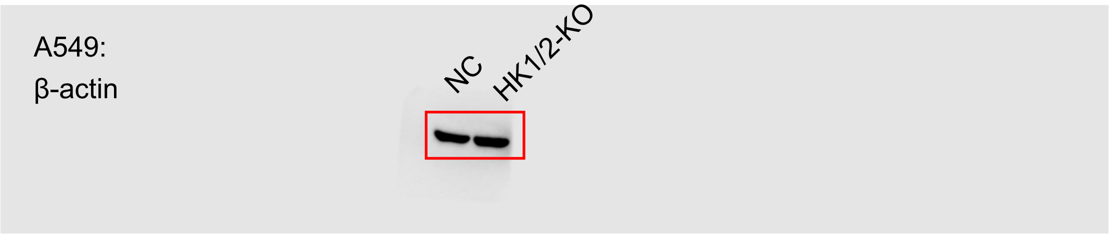
A549:
β-actin
NC
HK1/2-KO

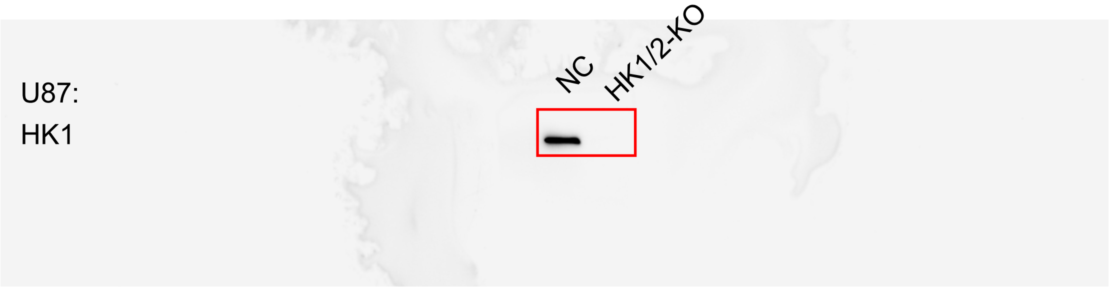
U87:
HK1
NC
HK1/2-KO

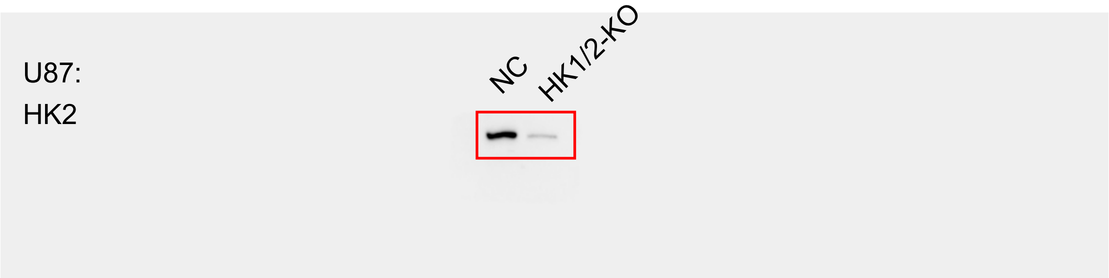
U87:
HK2
NC
HK1/2-KO

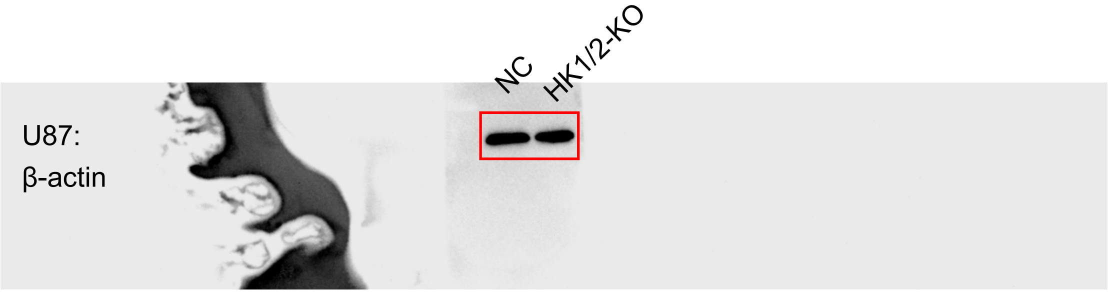
U87:
β-actin
NC
HK1/2-KO
